# Compaction of RNA Duplexes in the Cell**

**DOI:** 10.1002/anie.202009800

**Published:** 2020-10-13

**Authors:** Alberto Collauto, Sören von Bülow, Dnyaneshwar B. Gophane, Subham Saha, Lukas S. Stelzl, Gerhard Hummer, Snorri T. Sigurdsson, Thomas F. Prisner

**Affiliations:** ^1^ Institute of Physical and Theoretical Chemistry and Center of Biomolecular Magnetic Resonance Goethe University Frankfurt Max-von-Laue-Str. 7 60438 Frankfurt am Main Germany; ^2^ Department of Theoretical Biophysics Max Planck Institute of Biophysics Max-von-Laue-Str. 3 60438 Frankfurt am Main Germany; ^3^ Department of Chemistry Science Institute University of Iceland Dunhagi 3 107 Reykjavík Iceland; ^4^ Institute for Biophysics Goethe University Frankfurt Max-von-Laue-Str. 9 60438 Frankfurt am Main Germany

**Keywords:** EPR spectroscopy, molecular dynamics, PELDOR/DEER spectroscopy, RNA structures, site-directed spin labeling

## Abstract

The structure and flexibility of RNA depends sensitively on the microenvironment. Using pulsed electron‐electron double‐resonance (PELDOR)/double electron‐electron resonance (DEER) spectroscopy combined with advanced labeling techniques, we show that the structure of double‐stranded RNA (dsRNA) changes upon internalization into *Xenopus lævis* oocytes. Compared to dilute solution, the dsRNA A‐helix is more compact in cells. We recapitulate this compaction in a densely crowded protein solution. Atomic‐resolution molecular dynamics simulations of dsRNA semi‐quantitatively capture the compaction, and identify non‐specific electrostatic interactions between proteins and dsRNA as a possible driver of this effect.

## Graphical abstract

A combination of advanced labeling techniques for nucleic acids and in‐cell pulsed electron‐electron double‐resonance spectroscopy reveals compaction of the dsRNA A‐helix inside *Xenopus lævis* oocytes compared to the dilute solution. Atomic‐resolution molecular dynamics simulations in a dense protein solution semi‐quantitatively capture the compaction, pointing to the molecular interactions involved.

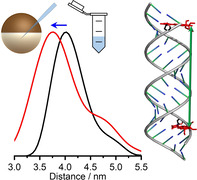

The biological function of RNA is tightly related to its structure and flexibility, which are both sensitive to the conditions and microenvironments present in the cell.^[1]^ For example, a general loss of RNA structure has been reported in cells, compared to in vitro conditions, while specific regions have been shown to be highly structured inside cells,[^2^, ^3^, ^4^] in some cases adopting folds that could not be replicated outside the cell.^[5]^ However, it is not known whether the intracellular environment also affects the structure of fundamental RNA motifs such as double‐stranded A‐form helices with Watson–Crick base pairs, with effects that can propagate to larger and more complex structures.^[6]^ Addressing such questions requires techniques that have sufficient resolution for the detection of subtle structural changes.

The structure of RNAs inside the cell has been deduced primarily from chemical probing experiments, which have achieved single‐nucleotide resolution for base‐pairing interactions in whole‐cell measurements.[^2^, ^3^, ^7^] Spectroscopic methods promise even higher resolution, albeit at a smaller scale. Nuclear magnetic resonance (NMR) spectroscopy is a valuable technique to investigate in a non‐invasive way the local structure, dynamics and interactions of nucleic acids inside cells.[^8^, ^9^, ^10^, ^11^, ^12^, ^13^] However, this approach is currently not suitable for probing long‐range structural changes at Ångstrom resolution on nucleic acids inside cells, information that could assist in detecting even small conformational rearrangements between structures inside and outside of the cell. As a reporter on long‐range structural dynamics, Förster resonance energy transfer (FRET) has provided promising results on the intracellular folding of an RNA hairpin.^[14]^ FRET can discriminate between structural models, but the determination of in‐cell‐derived distances with high precision remains challenging.^[15]^

Pulsed electron‐electron double‐resonance (PELDOR)/ double electron‐electron resonance (DEER) spectroscopy,[^16^, ^17^, ^18^] an electron paramagnetic resonance (EPR)‐based technique, is particularly suitable for probing conformational changes of nucleic acids by measuring long‐range distances at Ångstrom resolution between site‐specifically introduced paramagnetic tags (spin labels). The use of spin labels is an advantage for in‐cell studies because it suppresses the background from the other constituents of the cell. PELDOR benefits from a parameter‐free processing of the experimental data to yield distance probability distributions.[^19^, ^20^] PELDOR measurements of nucleic acids using rigid nitroxide spin labels can give insights into details of conformational ensembles,^[21]^ even resolving subtle differences within duplexes.[^22^, ^23^] Here we show, using PELDOR measurements, that the structure of an A‐form RNA duplex differs inside and outside cells. Molecular dynamics (MD) simulations of double‐stranded RNA (dsRNA) in a concentrated protein solution captured semi‐quantitatively the experimentally observed RNA compaction and point to the molecular interactions involved.

Early in‐cell PELDOR experiments on nucleic acids[^24^, ^25^] and proteins^[26]^ have been hampered by the limited stability of conventional paramagnetic tags in the reducing intracellular environment.^[27]^ This has motivated the development of several classes of reduction‐resistant spin labels based on substituted nitroxides,^[27]^ Gd^III^ chelates[^28^, ^29^, ^30^, ^31^] or trityl radicals,[^32^, ^33^, ^34^, ^35^] for which successful in‐cell distance measurements have been demonstrated.[^28^, ^36^, ^37^, ^38^, ^39^, ^40^, ^41^, ^42^, ^43^, ^44^, ^45^, ^46^, ^47^, ^48^] Here we chose a nitroxide functional group that is flanked by *gem*‐diethyl substituents to sterically shield the spin label from reduction.[^27^, ^49^, ^50^, ^51^, ^52^, ^53^, ^54^, ^55^, ^56^] Another important criterion in the selection of a spin label for PELDOR experiments is to limit the flexibility of the tether between the radical and the nucleic acid in order to increase the accuracy of the measurements. Therefore, we prepared the reduction‐resistant spin label, E^Im^Um (Figure 1 A and Scheme S1 in the Supporting Information), which is semi‐rigid due to an intramolecular hydrogen bond between the benzimidazole N‐H and O4 of the uracil, which restricts rotation around the single bond connecting the benzimidazole‐derived nitroxide to the nucleobase.[^57^, ^58^, ^59^, ^60^] E^Im^Um was converted to its phosphoramidite, incorporated into RNA using solid‐phase synthesis, the RNA characterized (Table S1 and Figure S1 in the Supporting Information) and the stability of the shielded nitroxide inside cells verified (Figure S2B in the Supporting Information).

**Figure 1:**
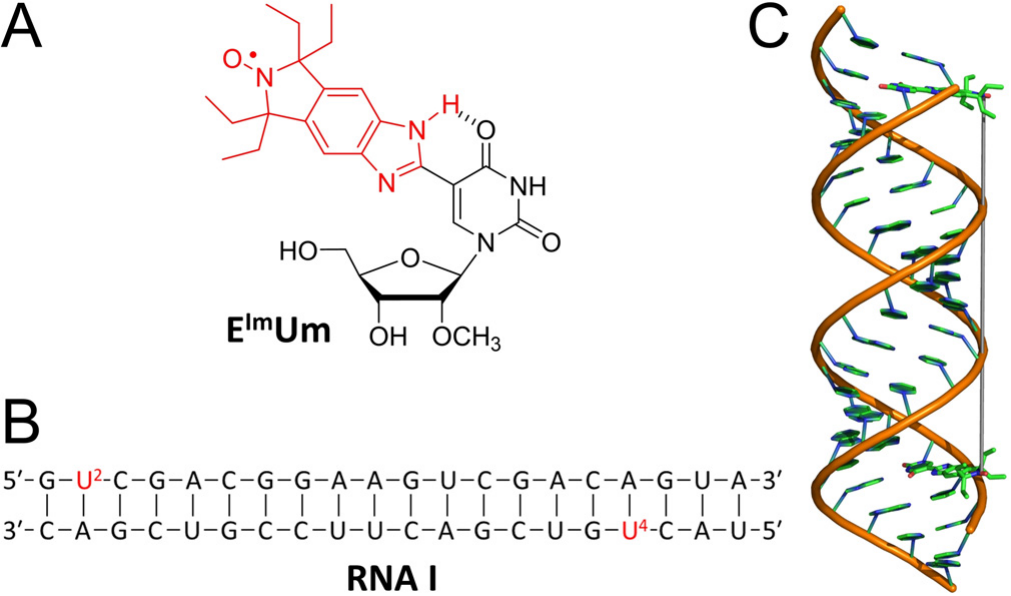
A) Structure of the E^Im^Um spin label. B) Sequence of the 20‐mer duplex RNA I; the spin‐labeled nucleotides are displayed in red. C) Model of the duplex RNA I containing the two E^Im^Um labels; the vertical line connects the nitroxide groups of the two spin labels.

The Q‐band PELDOR measurements on duplex RNA I (Figure 1 B) in a buffered solution were analyzed using a model‐free approach,^[61]^ yielding a distance probability distribution centered at 4.0 nm (Figure 2, black traces). For in‐cell PELDOR experiments, RNA I was introduced into the cytoplasm of *Xenopus lævis* oocytes by microinjection (see Supporting Information). The in‐cell Q‐band PELDOR data (Figure 2, red traces) show a clear and reproducible reduction of the inter‐spin distance by approximately 0.3 nm relative to the buffered solution. The statistical relevance of this shift is supported by four independent repeats on different in‐cell samples (multiple red traces in Figure 2 B) and by the robustness of the resulting distance probability distributions with respect to the analysis procedure (Figure S5 in the Supporting Information).

**Figure 2:**
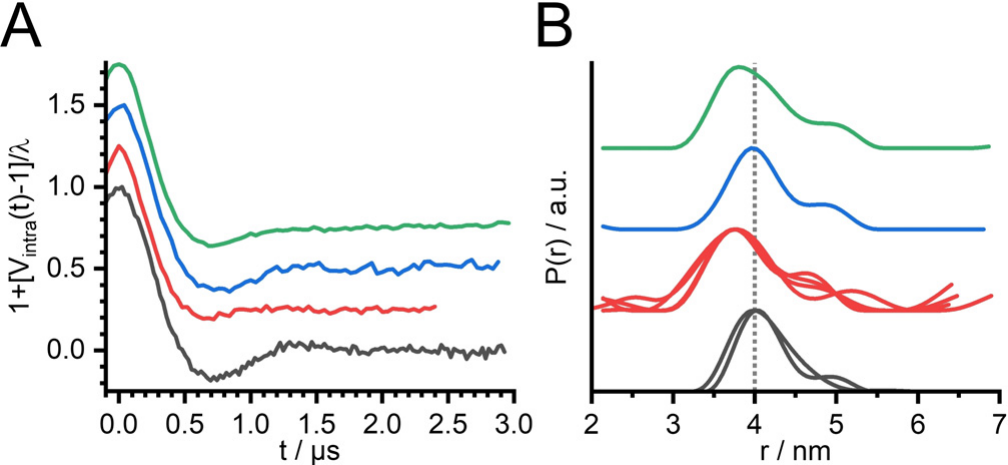
Q‐band 4‐pulse PELDOR background‐subtracted data normalized by the modulation depth (A) and the corresponding distance probability distributions obtained by model‐free analysis (B) for the duplex RNA I in a buffered solution (10 mM phosphate pH 7.0, 100 mM NaCl, 0.1 mM EDTA; black traces), in *Xenopus lævis* oocytes (red traces), in cytoplasmic extract (blue traces), and in a 200 mg mL^−1^ lysozyme solution (green traces). Multiple traces, where present, show results obtained from different samples. The original traces are reported in the Supporting Information (Figure S4) together with a validation of the distance probability distributions (Figure S5), confirming the statistical relevance of the reduction of the inter‐spin distance upon in‐cell internalization.

Moreover, in‐cell samples are characterized by a broader distance distribution (Figure 2 B), consistent with a faster damping of the oscillations of the PELDOR traces (Figure 2 A).

Because of the restricted mobility of the spin label with respect to the backbone of the RNA, this broadening can be assigned to a more heterogeneous conformational ensemble of the duplex inside the cells.

Both the reduction of the inter‐spin distance and the increased damping of the dipolar oscillations were reproduced with X‐band PELDOR measurements (Figure S6,S7 in the Supporting Information).

In an attempt to identify the origin of these in‐cell structural changes in RNA I, we incubated the labeled nucleic acid with a cytoplasmic extract, obtained by centrifugation of the oocytes (see Supporting Information). Remarkably, the PELDOR data for this sample (Figure 2, blue trace) did not show any relevant differences with respect to the in vitro experiments, likely due to the dilution factor of the extract (see Supporting Information). In contrast, the PELDOR data for the duplex in a concentrated solution of the protein lysozyme (LYZ) (Figure 2, green trace) showed a similar inter‐spin distance as the in‐cell samples. We hypothesized that lysozyme was acting as a positively charged crowder that bound nonspecifically to RNA, supported by native gel electrophoresis (Figure S11 in the Supporting Information). Using bovine serum albumin as an alternative crowder revealed no relevant changes in the PELDOR data relative to the buffered solution (Figure S4 in the Supporting Information), ruling out nonelectrostatic crowding effects. These experimental findings likely reflect interactions of the exogenous RNA in the cell with RNA‐binding proteins, which are abundant in the cytoplasm of *Xenopus lævis* oocytes.[^62^, ^63^, ^64^] Specific protein binding to dsRNA helices is typically mediated through aromatic and basic residues.[^65^, ^66^] Here, the results of the gel electrophoresis experiments combined with the MD simulations showed that dsRNA is also subject to strong non‐specific electrostatic interactions in a dense solution of basic proteins.

To rule out the possibility that the spin label played an explicit role on the detected change of the inter‐spin distance, and/or that these effects were sequence specific, additional experiments were performed on 24‐mer RNA duplexes labeled with the tetraethyl‐shielded, thiourea‐based spin label E‐TU^[53]^ (Figure S8 and S9,S10 in the Supporting Information). The results were in line with the trend observed in Figure 2, namely a reproducible shortening of the inter‐spin distances for the in‐cell and lysozyme‐containing samples. Thus, our results are consistent with a genuine conformational change of dsRNA upon internalization into cells.

The long‐range distance change between two labels in the RNA duplex is not sufficient by itself to shed light on the microscopic origin of the RNA conformational change. To gain additional insights, we performed MD simulations of the duplex RNA free in an aqueous solution and in the presence of a high concentration of lysozyme. The simulations were designed to give us a molecular view of nonspecific dsRNA‐protein interactions and an understanding of their effects on dsRNA structure.

Realistic atomistic simulations of mixed‐component systems such as RNA and proteins pose a challenging problem, because the molecular interactions between RNA, proteins, water and ions all need to be balanced. We found that the commonly employed RNA force field OL3[^67^, ^68^, ^69^] in combination with the dispersion‐corrected TIP4P‐D water model^[70]^ (known to slightly improve the solvation accuracy of ssRNA^[71]^) preserved the A‐helix structure of dsRNA in solution (Figure S12 in the Supporting Information). We combined the RNA and water force fields with the Amber99sb*‐ILDN‐q protein force field, which has been shown to describe concentrated solutions of lysozyme and other proteins very well,^[72]^ including nonspecific protein‐protein binding, protein diffusion, and bulk viscosity. In microsecond‐scale (590 ns to 2.8 μs; see Table S3 in the Supporting Information) atomistic MD simulations of crowded solutions with 20 lysozyme proteins at a protein concentration of 200 mg mL^−1^, we aimed to capture the effects of nonspecific protein‐RNA interactions on the structure and stability of the dsRNA A‐helix (Movie S1 in the Supporting Information).

Simulations of the dsRNA in dense lysozyme solution showed that the RNA retains its helical structure (Figure 3 and S13, S14 in the Supporting Information) while undergoing compaction up to 1 Å relative to dilute solution, as seen in the average structures (Figure 3 C) and decreased base‐base distances (Figure 3 E). This compaction is comparable in magnitude to the 3‐Å contraction seen in the PELDOR experiments in lysozyme and in cell. The MD simulations show diverse lysozyme‐RNA binding modes, reflecting the nonspecific character of this interaction (Figure S15 in the Supporting Information). In particular, we found strong and persistent interactions between the negatively charged phosphate groups of the RNA backbone with positively charged amino acids of lysozyme, in particular arginine (Figure 3 B). Our simulation results suggest that the nonspecific reduction in the effective charge of the backbone by the counter‐charges on the proteins and the formation of tight interactions with multiple amino acids simultaneously (Figure 3 B) are drivers of the RNA compaction.

**Figure 3:**
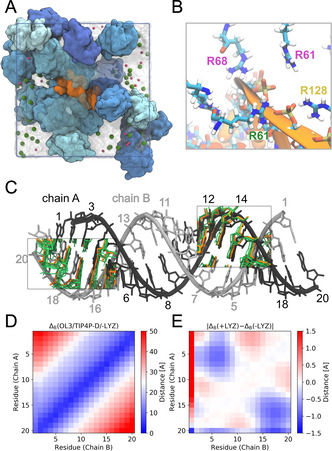
RNA conformational dynamics in crowded LYZ solution as revealed by atomistic MD simulations with fully flexible RNA and proteins in explicit solvent. A) Simulation snapshot showing the RNA A‐helix (orange) in crowded LYZ solution (blue shades) at 200 mg mL^−1^ concentration. Na^+^ and Cl^−^ ions are shown as pink and green spheres. Water is indicated by the transparent surface. B) Zoom‐in on the MD simulation snapshot showing basic residues interacting with the phosphate backbone of the RNA. Arginine labels are colored to distinguish the proteins they belong to. C) Compaction of the RNA. Reference idealized A‐helix structure (gray) and mean structures from simulations of the RNA in dilute (orange; absence of LYZ) and dense solution (green; 200 mg mL^−1^ LYZ) are compared for bases separated by about one helical turn. D) Average base‐base distances in the MD simulations of the dsRNA in dilute solution. E) Difference in the average base‐base distances in presence and absence of LYZ.

In summary, in‐cell PELDOR experiments, enhanced by the highly performant E^Im^Um spin label, revealed a subtle compaction of the structure of RNA duplexes in oocytes. MD simulations of complex protein‐RNA mixtures at atomic resolution demonstrated how interactions with positively charged proteins slightly compact the RNA, and provide a possible structural explanation for the mean compaction demonstrated independently by the PELDOR measurements. It will be interesting to further investigate our findings for other cell types and states, and to correlate them to proteomic data on the abundance of basic and/or RNA‐binding proteins.

## Conflict of interest

The authors declare no conflict of interest.

## Supporting information

As a service to our authors and readers, this journal provides supporting information supplied by the authors. Such materials are peer reviewed and may be re‐organized for online delivery, but are not copy‐edited or typeset. Technical support issues arising from supporting information (other than missing files) should be addressed to the authors.

Supplementary

Supplementary
