# Supplementary material for: Compaction of RNA Duplexes in the Cell
Source: Angew Chem Int Ed Engl. 2020 Oct 13;59(51):23025–9. doi: 10.1002/anie.202009800 (PMC7756485; doi:10.1002/anie.202009800)
Supplement: Supplementary file 1 — Supplementary [file ANIE-59-23025-s001.pdf]

Angewandte Chemie
Eine Zeitschrift der Gesellschaft Deutscher Chemiker

Supporting Information

Compaction of RNA Duplexes in the Cell**

Alberto Collauto, Sören von Bülow, Dnyaneshwar B. Gophane, Subham Saha, Lukas S. Stelzl, Gerhard Hummer, Snorri T. Sigurdsson,* and Thomas F. Prisner**

anie_202009800_sm_miscellaneous_information.pdf
anie_202009800_sm_SI_MovieS1.mp4

Table of Contents

General materials and methods ... 3
Preparation of RNA containing the $E^{lm}Um$ nitroxide ... 3
Preparation of RNA containing the E-TU nitroxide ... 3
Synthesis of the nucleoside $E^{lm}Um$ and its corresponding phosphoramidite ... 4
MALDI-TOF MS analyses of oligoribonucleotides ... 19
Thermal denaturation experiments ... 20
List of the RNA duplexes ... 20
Preparation of the samples for *in vitro* EPR spectroscopy ... 21
Preparation of the cytoplasmic extract ... 21
Internalization of the RNA duplexes inside *Xenopus lævis* oocytes and preparation of the in-cell EPR samples ... 21
X-band room-temperature CW-EPR spectroscopy ... 22
Instrumentation and general experimental conditions for X- and Q-band pulsed EPR spectroscopy ... 23
Echo-detected field-sweep and 2-pulse echo decay experiments at X- and Q-band ... 23
Q-band PELDOR spectroscopy of RNA I ... 24
X-band PELDOR spectroscopy of RNA I ... 26
Q-band PELDOR spectroscopy of the E-TU-labeled RNA duplexes ... 28
Native polyacrylamide gel electrophoresis (PAGE) ... 30
Molecular dynamics simulations ... 31
References ... 36
Author Contributions ... 36

General materials and methods

All commercially available reagents were purchased from Sigma-Aldrich, Acros Organics or Fluka and used without further purification. 2′-O-methyluridine was purchased from Rasayan Inc. USA. All moisture- and air-sensitive reactions were performed in flame-dried glassware under a positive pressure of nitrogen. Solvents were distilled prior to use and stored over activated molecular sieves (4 Å) under nitrogen. Water was purified by a Milli-Q water purification system. Analytical thin layer chromatography (TLC) was performed on silica gel glass plates (Silicycle, ultra-pure silica gel, 60Å, F_{254}); TLC visualization was performed with UV light or by staining with *p*-anisaldehyde. Flash-column chromatography was performed on silica gel (Silicycle, 230-400 mesh, 60Å). ^{1}H-spectra were recorded using deuterated solvents as internal standards on a Bruker Advance 400 spectrometer; chemical shifts are reported in ppm. Residual proton signals from the deuterated solvents were used as references [d_6-DMSO (2.50 ppm), $CDCl_3$ (7.26 ppm)], for ^{1}H spectra. ^{13}C NMR chemical shifts are reported in reference to undeuterated residual solvent ($CDCl_3$ (77.0 ppm), d_6-DMSO (39.43 ppm). ^{31}P NMR chemical shift is reported relative to 85% H_3PO_4 as an external standard. Commercial grade $CDCl_3$ was passed over basic alumina shortly before use, to record the ^{1}H-NMR and ^{31}P-NMR of phosphoramidite **8**. Mass spectrometric analyses of all organic compounds were performed on an ESI-HRMS (Bruker, microTOF-Q) in positive or negative ion mode.
RNA oligonucleotides were synthesized on an automated ASM800 DNA/RNA synthesizer (Biosset, Novosibirsk, Russia) by using a trityl-off protocol and phosphoramidites with standard protecting groups on a 1.0 mmol scale, using 1000 Å CPG columns. All commercial phosphoramidites, CPG columns, and solutions were purchased from ChemGenes Corporation (Wilmington, MA). Upon completion of synthesis, the oligoribonucleotides were cleaved from the solid support and the nucleobases and phosphodiesters deprotected in a 1:1 mixture of conc. aqueous NH_3 and 8 M $MeNH_2$ in EtOH (2 mL) at 65 °C for 40 min. The supernatant was collected, the bead was washed three times with a mixture of EtOH:H_2O (1:1, 300 µL), and the combined washings were dried. The 2′-*O*-TBDMS groups were removed by treatment with a mixture of Et_3N•3HF:DMF (3:1, 800 µL) at 55 °C for 1.5 h, followed by addition of H_2O (200 µL). This mixture was transferred to a 50 mL Falcon tube and n-butanol (40 mL) was added and stored at -20 °C for 12 h, centrifuged and the solvent decanted from the RNA pellet. The crude RNA was subsequently purified by 20% denaturing polyacrylamide gel electrophoresis (DPAGE). The RNA oligonucleotide bands were visualized under UV light, excised from the gel, crushed, and eluted from the gel with a Tris buffer (2×10 mL; Tris (10 mM, pH 7.5), NaCl (250 mM), Na_2EDTA (1 mM)). The RNA elutions were filtered through a 0.45 mm cellulose acetate membrane (Whatman) and desalted using a Sep-Pak cartridge (Waters Corporation). The dried oligoribonucleotides were dissolved in sterile H_2O (400 µL) and their final concentrations were calculated according to Beer's law based on UV absorbance of oligoribonucleotides at 260 nm. Extinction coefficients were determined by using the UV WinLab oligoribonucleotide calculator (V2.85.04; Perkin Elmer). Molecular weights of oligoribonucleotides were determined by MALDI-TOF analysis (Bruker, Autoflex III) after calibration with an external standard. UV/vis spectra were recorded on a PerkinElmer Lambda 25 UV/vis spectrometer.

Preparation of RNA containing the E^{Im}Um nitroxide

E^{Im}Um was incorporated into RNA oligonucleotides by solid phase synthesis using the previously reported protocol,[1] with a slight modification. The activator 5-(benzylthio)-1*H*-tetrazole, usually used for RNA synthesis was not suitable for **E^{Im}Um** incorporation in oligoribonucleotides, as we observed a coupling efficiency of less than 5% (as judged by the color during trityl deprotection after coupling of **E^{Im}Um** phosphoramidite). Therefore, 5-(ethylthio)-1*H*-tetrazole, the activator generally used in our laboratory for DNA synthesis, was used instead. The spin-labeled phosphoramidite was incorporated manually into the oligoribonucleotides by pausing the synthesizer program after completion of the prior cycle, removing the column from the synthesizer, and running the standard activator solution (200 µL) and a solution of the spin-labeled phosphoramidite (0.05 M, 200 µL) prepared in 1,2-dichloroethane back and forth through the column for ~10–12 min. After manual coupling, the column was remounted on the synthesizer and the synthesis cycle was completed.

Preparation of RNA containing the E-TU nitroxide

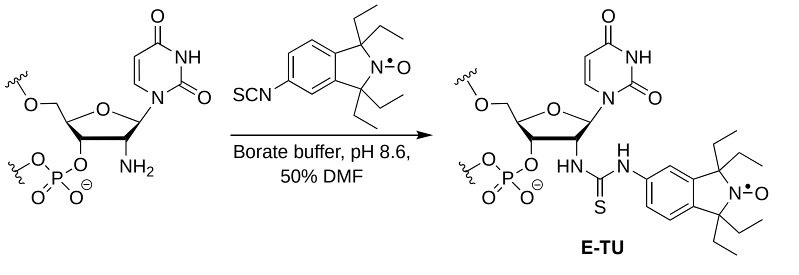

The procedure was essentially the same as previously described,[2] which entailed reacting the 2′-amino uridine-modified RNA with the isothiocyanate in borate buffer (100 mM, pH 8.6) containing DMF (50%) at 37 °C for 8 h. After EtOH precipitation, the spin-labeled product was purified by 20% DPAGE followed by another round of EtOH precipitation to obtain the E-TU-labeled RNA oligonucleotide.

Synthesis of the nucleoside E^lm^Um and its corresponding phosphoramidite

The synthesis of **E^lm^Um** (**Scheme S1**) began with conversion of 5-nitro-1,1,3,3-tetraethylisoindoline (**1**)[3] to 5-amino-6-nitro-1,1,3,3-tetraethylisoindoline (**2**) by direct amination using t-BuOK and 1,1,1 trimethylhydrazinium iodide in DMSO.[4] The nitro group of **2** was reduced to give 5,6-diamino 1,1,3,3-tetraethylisoindoline (**3**), which was reacted with aldehyde **4**[5] in presence of $K_3Fe(CN)_6$ to afford the isoindoline benzimidazole derivative of 2'-methoxyuridine (**5**). Benzimidazole **5** was oxidized with *m*-CPBA in the presence of NaN_3 to give nitroxide **6** in good yields. The TBDMS groups of **6** were removed to give **E^lm^Um**, which was sequentially tritylated to give compound **7** and phosphilated to give phosphoramidite **8**.

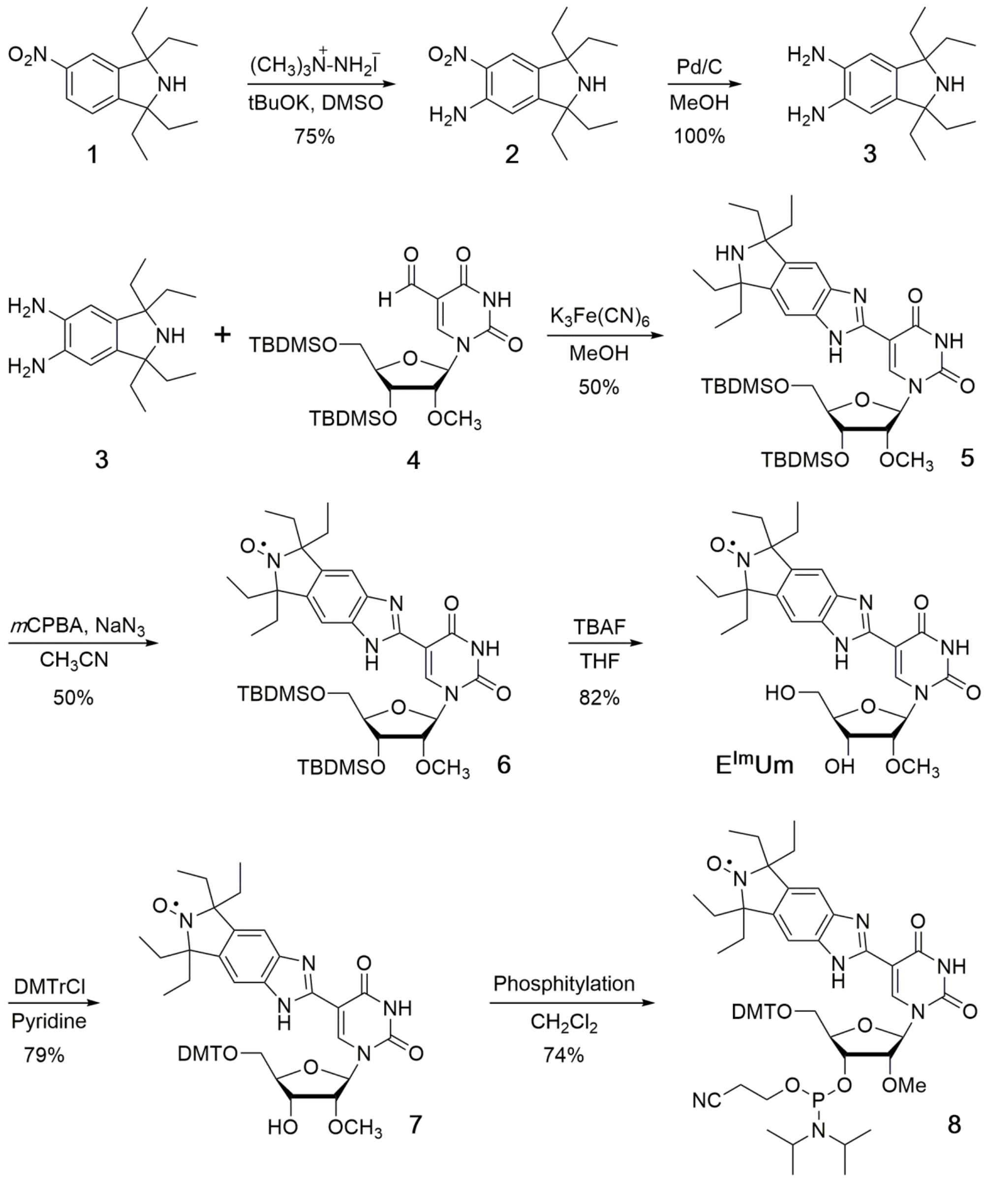

Scheme S1. Synthesis of the nucleoside **E^lm^Um** and its corresponding phosphoramidite (**8**).

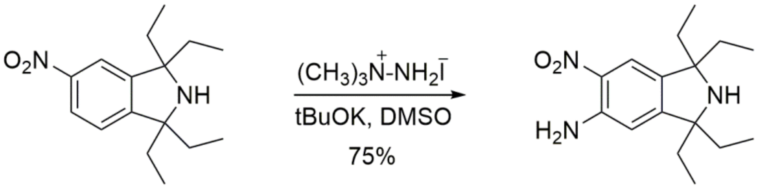

1   2

1,1,3,3-tetraethyl-6-nitroisoindolin-5-amine (2). To a solution of *N,N,N*-trimethylhydrazine iodide (5.06 g, 0.025 mol) in DMSO (20 mL) was added t-BuOK (2.81 g, 0.025 mol) and the solution stirred at 23 °C for 30 min. Compound **1** (3.15 g, 0.011 mol) was added and the reaction stirred for 40 h at 23 °C. The reaction mixture was poured into ice-cold water (150 mL), followed by extraction with CH_2Cl_2 (3 x 200 mL), the combined organic phases dried over anhydrous Na_2SO_4, filtered and concentrated *in vacuo*. The crude product was purified by flash silica gel column chromatography using a gradient elution (CH_2Cl_2:MeOH; 100:0 to 97:3) to give compound **2** as a yellow solid (2.50 g, 75% yield).

^{1}H NMR ($CDCl_3$): δ 7.79 (s, 1H), 6.46 (s, 1H), 6.04 (s, 2H), 1.88 – 1.54 (m, 8H), 0.87 (t, *J* = 7.4 Hz, 12H).

^{13}C NMR ($CDCl_3$): δ 157.80, 144.71, 138.35, 132.26, 119.79, 111.69, 68.48, 67.69, 33.94, 33.78, 9.02, 8.97.

HR-ESI-MS $(M + H)^+$: calcd. for $C_{16}H_{26}N_3O_2$ 292.2020, found 292.2017.

^{1}H NMR spectrum of 2

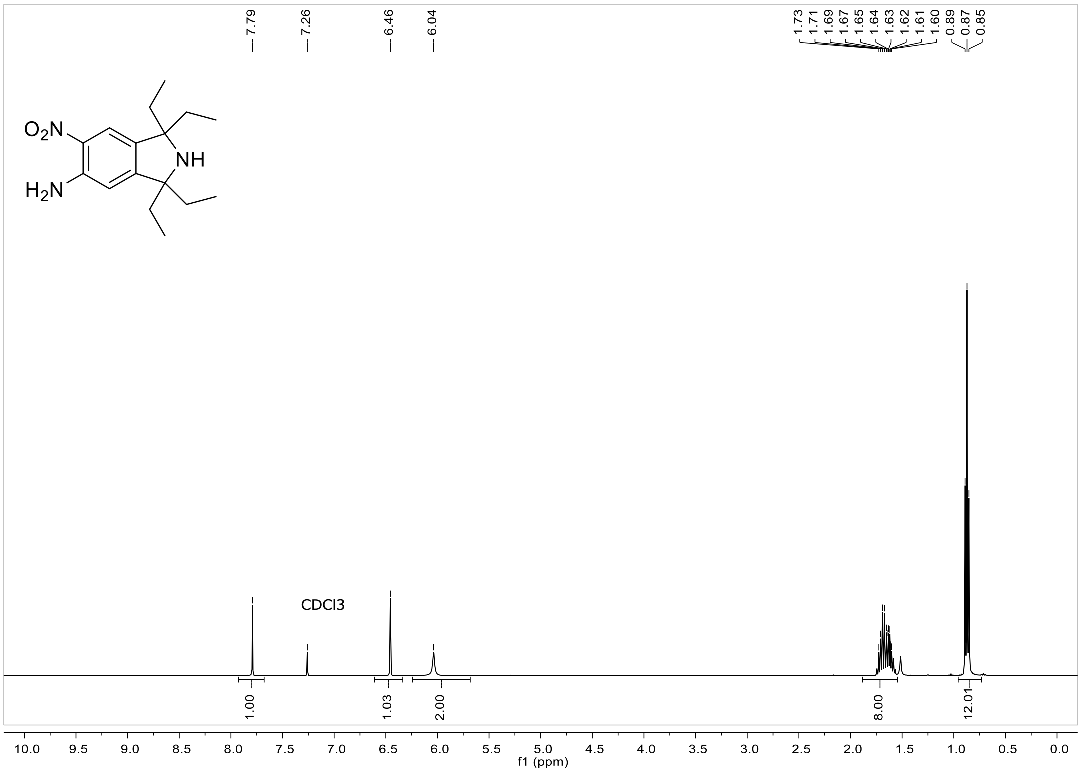

^{13}C NMR spectrum of 2

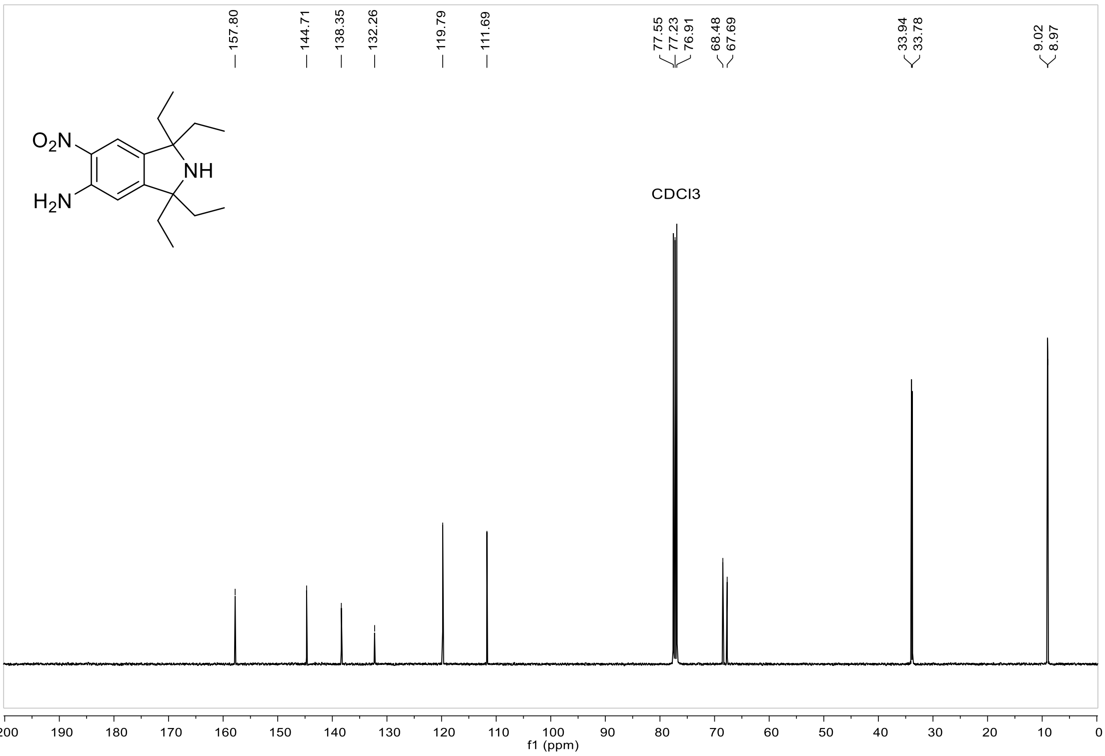

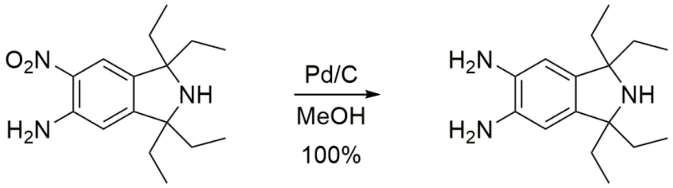

2 3

1,1,3,3-tetraethylisoindoline-5,6-diamine (3). To a solution of compound **2** (400.0 mg, 1.37 mmol) in MeOH (16 mL) was added 10% Pd/C (40.0 mg) and the mixture was hydrogenated at 55 psi hydrogen pressure at 23 °C for 2 h. The reaction mixture was filtered through a pad of celite and the filtrate was concentrated *in vacuo* to yield 1,1,3,3-tetraethylisoindoline-5,6-diamine (358.0 mg, 100%), which was used in the next reaction without further purification.

^{1}H NMR ($CDCl_3$): δ 6.40 (s, 2H), 3.75 – 2.54 (m, 4H), 1.64 (dtd, J = 21.3, 13.8, 7.3 Hz, 8H), 0.86 (t, J = 7.5 Hz, 12H).

^{13}C NMR ($CDCl_3$): δ 139.67, 133.90, 110.92, 68.25, 34.04, 9.18.

HR-ESI-MS $(M + H)^+$: calcd. for $C_{16}H_{28}N_3$ 262.2278, found 262.2098.

^{1}H NMR spectrum of 3

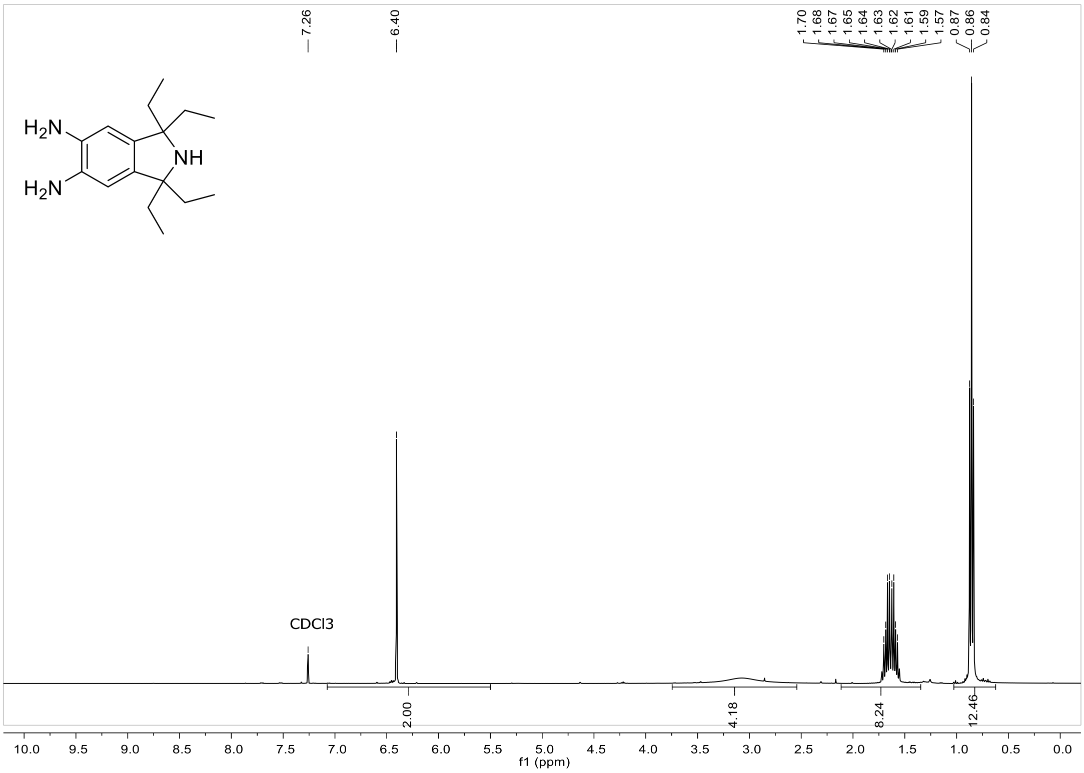

^{13}C NMR spectrum of 3

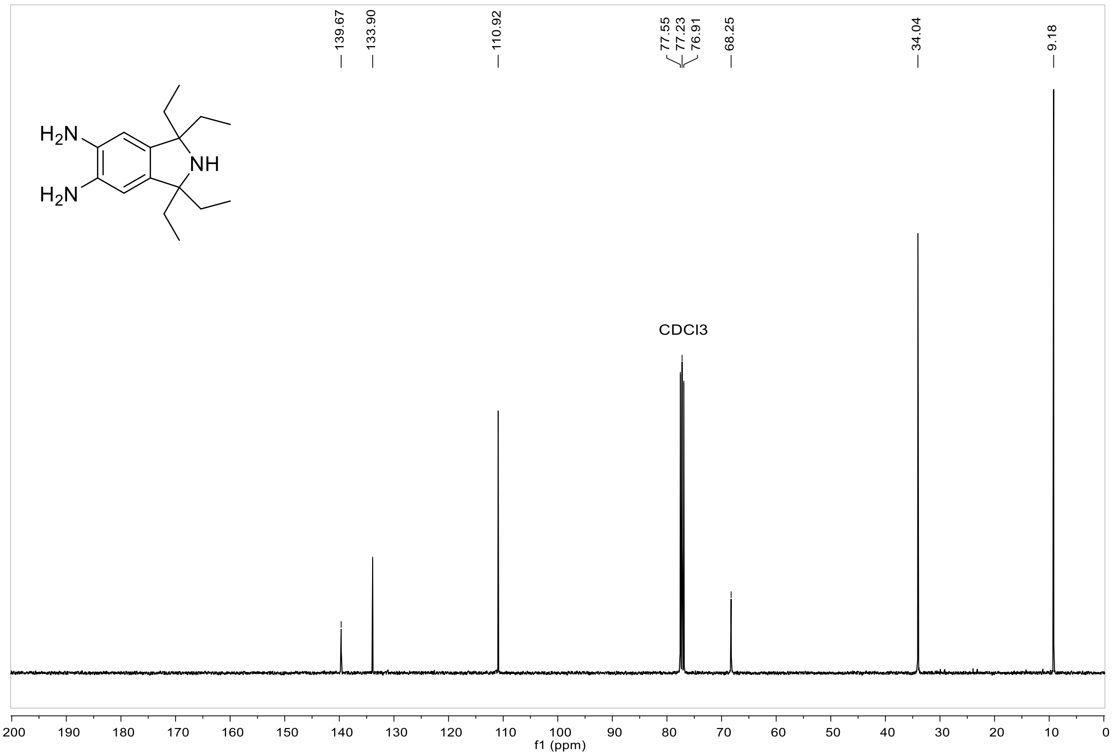

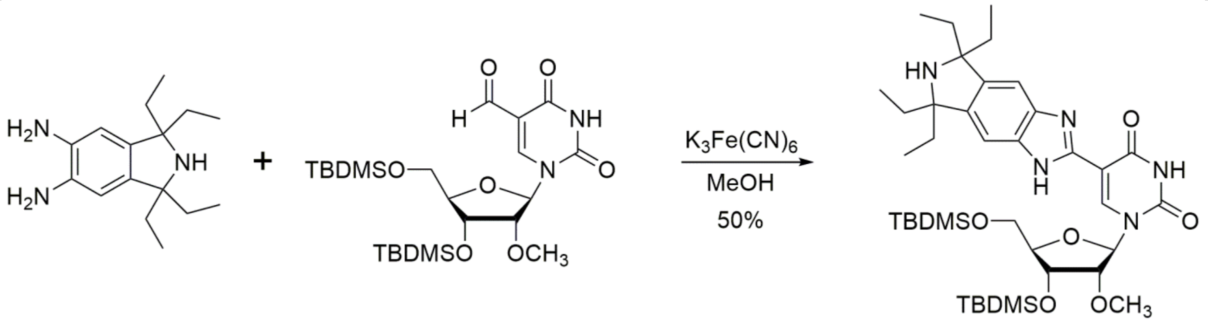

3 4 5

Nucleoside 5. To a solution of compound **4**[5] (705.0 mg, 1.37 mmol) and 1,1,3,3- tetraethylisoindoline-5,6-diamine (**3**) (358.0 mg, 1.37 mmol) in MeOH (15 mL) was added $K_3Fe(CN)_6$ (541.0 mg, 1.64 mmol) and the resulting solution was stirred for 16 h at 23 °C. The solution was concentrated *in vacuo* and the crude product was purified by flash silica gel column chromatography using a gradient elution (CH_2Cl_2:MeOH; 100:00 to 98:02) to give **5** as a yellow solid (520.0 mg, 50% yield).

^{1}H NMR (d_6-DMSO): δ 11.99 (s, 1H), 8.58 (s, 1H), 7.24 (s, 1H), 7.15 (s, 1H), 5.93 (d, *J* = 4.4 Hz, 1H), 4.27 (t, *J* = 4.9 Hz, 1H), 4.03 – 3.84 (m, 3H), 3.84 – 3.59 (m, 1H), 3.38 (s, 3H), 1.64 (td, *J* = 13.9, 6.4 Hz, 8H), 0.90 – 0.75 (m, 31H), 0.14 (d, *J* = 2.6 Hz, 6H), 0.10 (t, *J* = 2.5 Hz, 6H).

^{13}C NMR (d_6-DMSO): δ 161.76, 149.57, 145.35, 142.19, 142.11, 141.55, 139.73, 133.94, 110.74, 104.95, 104.89, 87.05, 84.68, 82.28, 69.56, 66.94, 66.77, 62.13, 57.70, 45.68, 33.94, 33.87, 26.05, 25.95, 25.59, 18.08, 17.77, 11.52, 8.85, 8.79, 8.77, -4.78, -5.01, -5.42, -5.44.

HR-ESI-MS $(M + H)^+$: calcd. for $C_{39}H_{66}N_5O_6Si_2$ 756.4546, found 756.4584.

^{1}H NMR spectrum of 5

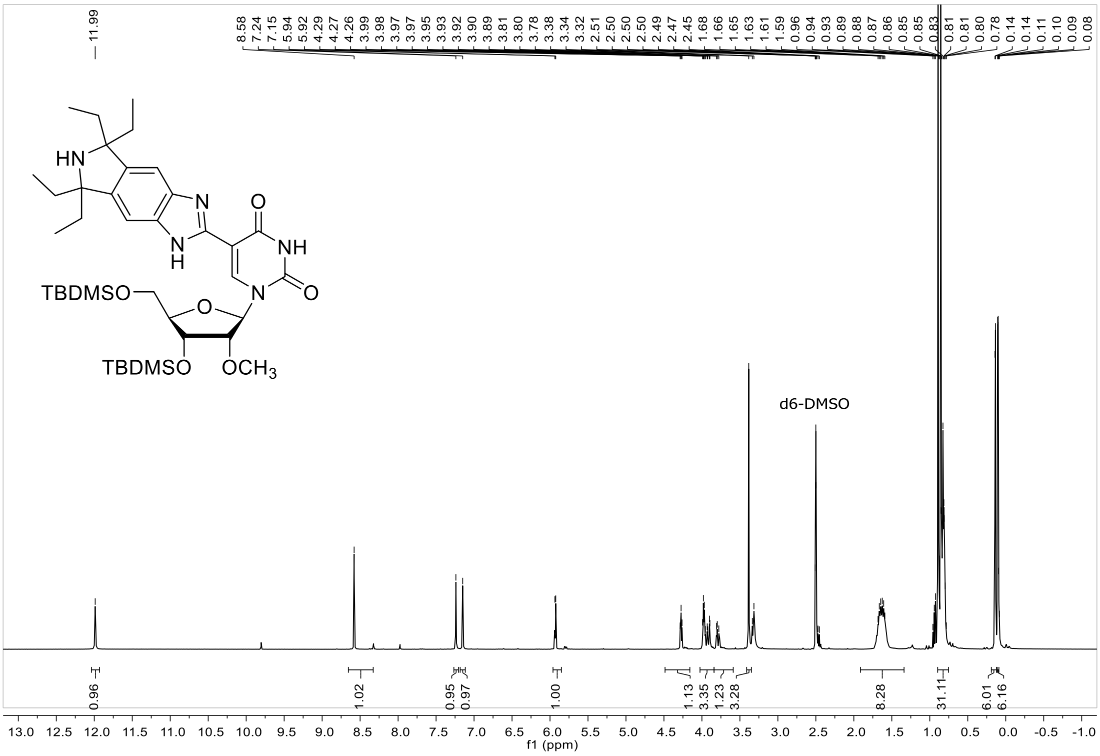

^{13}C NMR spectrum of 5

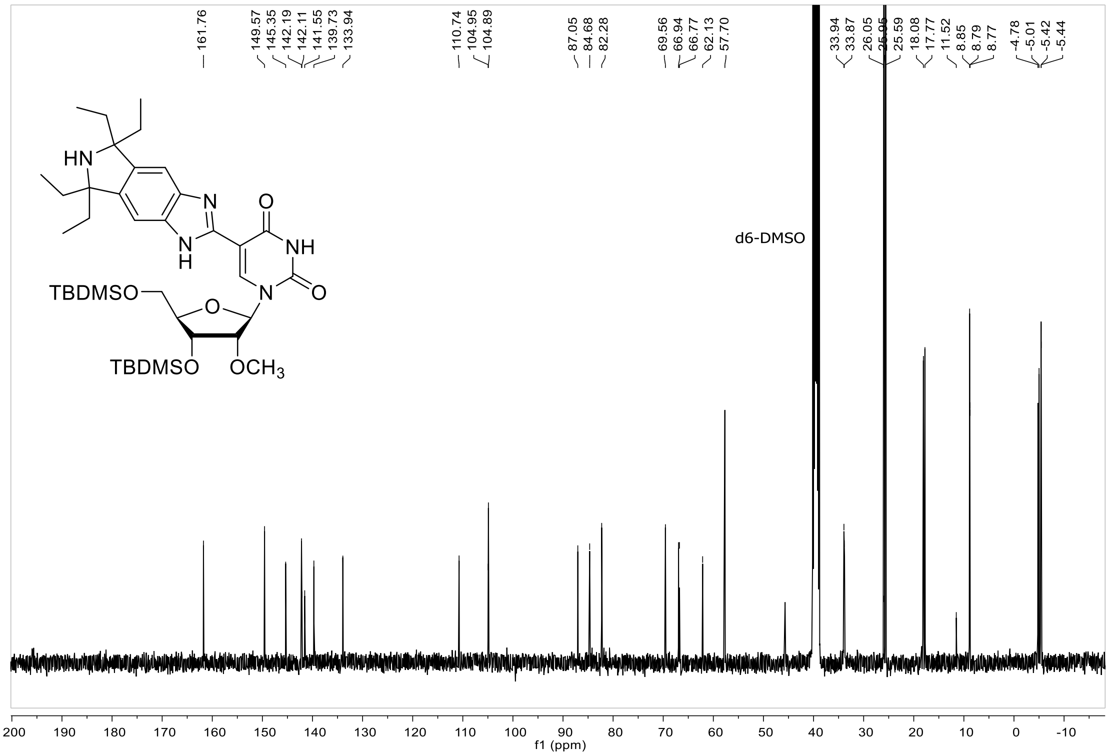

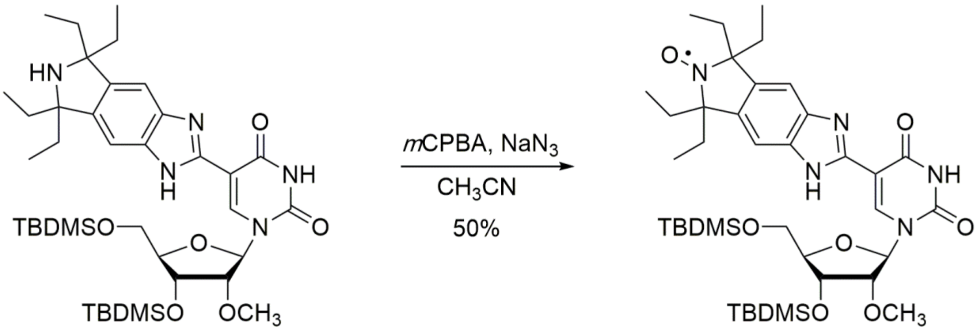

5   6

Nitroxide 6. To a suspension of **5** (490.0 mg, 0.6486 mmol) in CH_3CN (20 mL) was added NaN_3 (169.0 mg, 2.594 mmol) and the mixture was stirred at 23 ºC. After 30 min, *m*CPBA (224.0 mg, 1.297 mmol) was added. After further stirring for 16 h, the reaction mixture was concentrated *in vacuo* and the residue was purified by silica gel column chromatography using a gradient elution (CH_2Cl_2:MeOH; 100:0 to 98:2) to give compound **6** as a yellow solid (252.0 mg, 50% yield).

^{1}H NMR ($CDCl_3$): δ 11.46 (br s), 10.08 (br s), 8.90 (br s), 8.57 (br s), 8.33 (br s), 8.16 (br s), 8.02 (br s), 7.60 (br s), 6.20 (br s), 6.11 (br s), 5.79 (br s), 4.44 (br s), 4.18 (br s), 4.05 (br s), 3.54 (br s), 1.00 (br s), 0.86 (br s), 0.26 (br s), 0.19 (br s).

^{13}C NMR ($CDCl_3$) δ 184.22, 162.26, 162.12, 161.31, 149.65, 147.92, 140.41, 133.58, 133.35, 132.49, 129.24, 128.68, 128.58, 126.99, 126.83, 102.65, 87.17, 84.80, 81.92, 68.70, 68.22, 62.36, 61.30, 57.19, 24.87, 24.63, 24.59, 24.46, 24.40, 24.37, 17.16, 16.79, -5.88, -5.96, -6.06, -6.58, -6.74.

HR-ESI-MS $(M + H)^+$: calcd. for $C_{39}H_{66}N_5O_7Si_2$ 771.4417, found 771.4392.

SUPPORTING INFORMATION

^{1}H NMR spectrum of 6

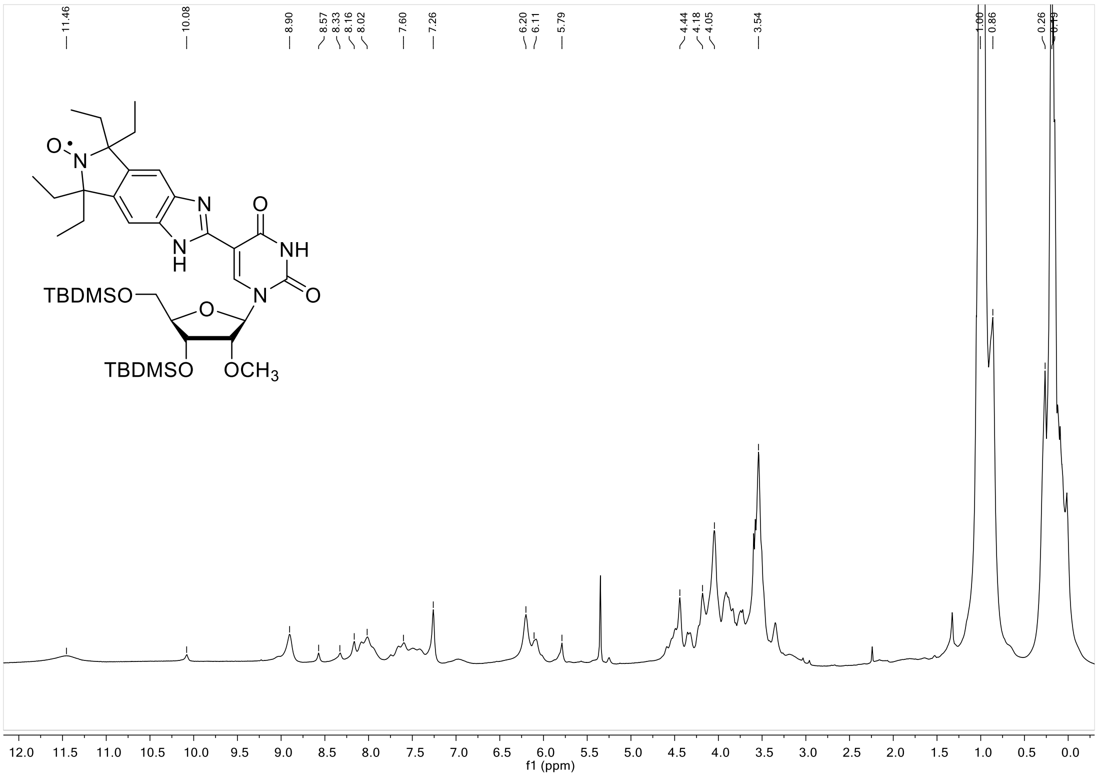

^{13}C NMR spectrum of 6

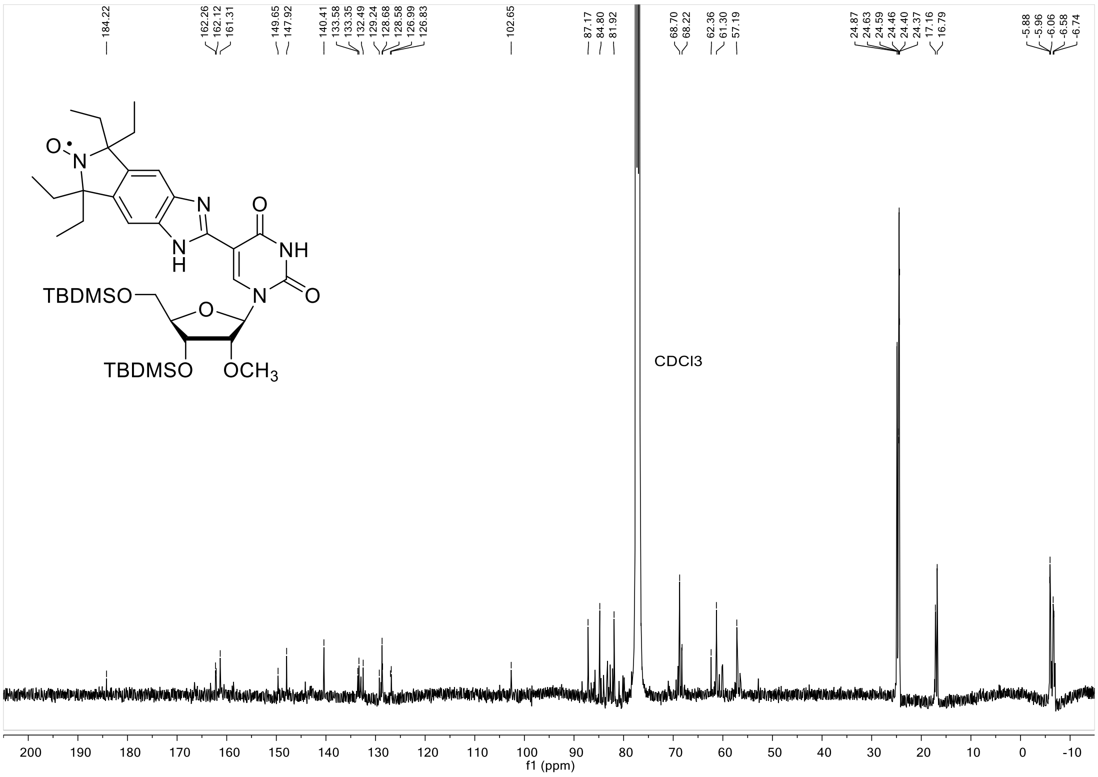

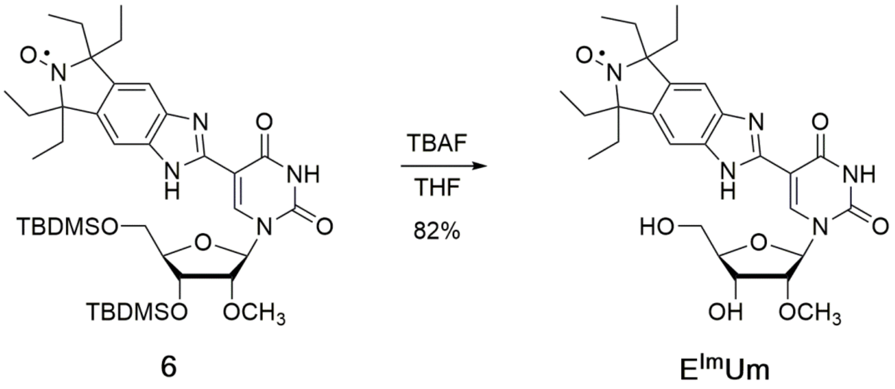

Spin-labeled nucleoside E^{Im}Um. A solution of nitroxide **6** (150.0 mg, 0.1945 mmol) in THF (15 mL) was added *tert*-butyl ammonium fluoride (0.450 mL, 1 M, 95%, 0.4279 mmol) and the solution stirred at 23 °C for 16 h. The solvent was removed *in vacuo* to give a sticky reddish oil. The crude product was purified by flash column chromatography (silica gel) using a gradient elution (CH_2Cl_2:MeOH; 100:0 to 95:5) to give compound **E^{Im}Um** as a yellow solid (84 mg, 80% yield).

^{1}H NMR ($CDCl_3$) δ 9.88 (br s), 9.34 (br s), 6.22 (br s), 5.68 (br s), 5.34 (s), 4.13 (br s), 3.93 (br s), 3.63 (br s), 3.07 (br s), 2.10 (br s), 1.99 (br s), 1.35 (br s), 0.95 (br s), 0.15 (br s).

^{13}C NMR ($CDCl_3$) δ 161.40, 148.20, 141.39, 102.29, 97.36, 87.58, 84.80, 82.61, 67.72, 66.40, 59.85, 58.31, 52.96, 32.39, 28.54, 24.70, 24.63, 24.04, 22.49, 19.08, 16.98, 12.99, 12.53.

HR-ESI-MS $(M + H)^+$: calcd. for $C_{27}H_{37}N_5O_7$ 543.2693, found 543.2646.

^{1}H NMR spectrum of E^{Im}Um

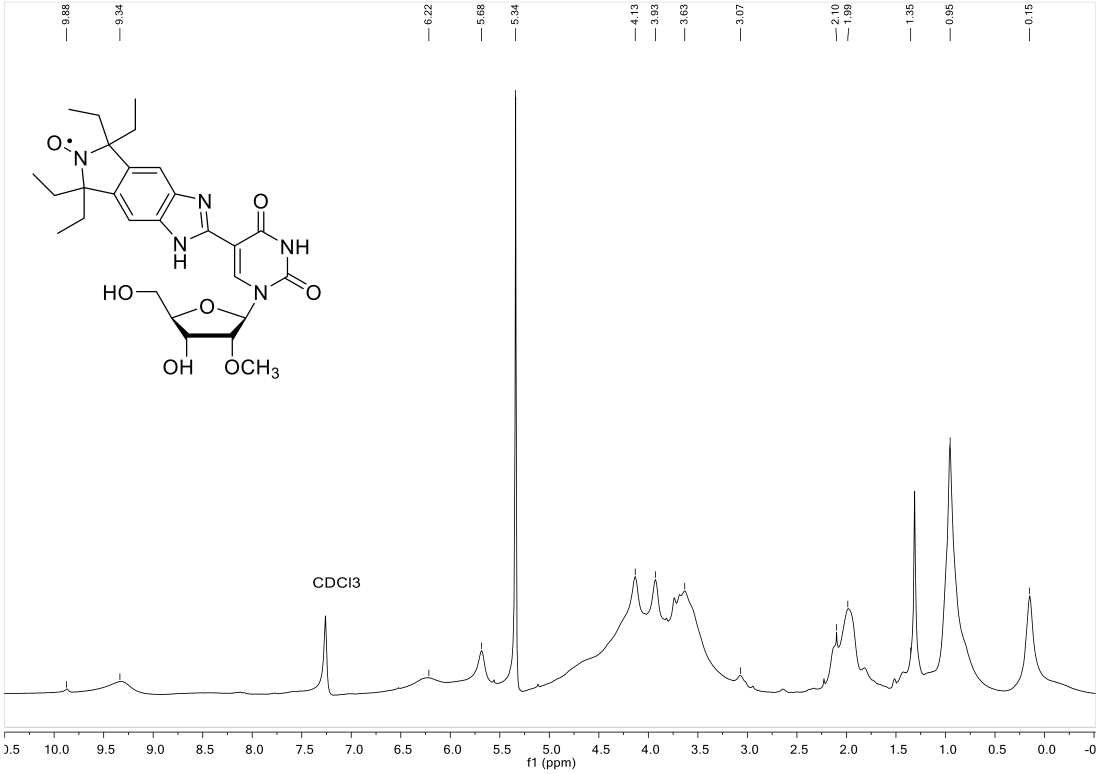

^{13}C NMR spectrum of E^{Im}Um

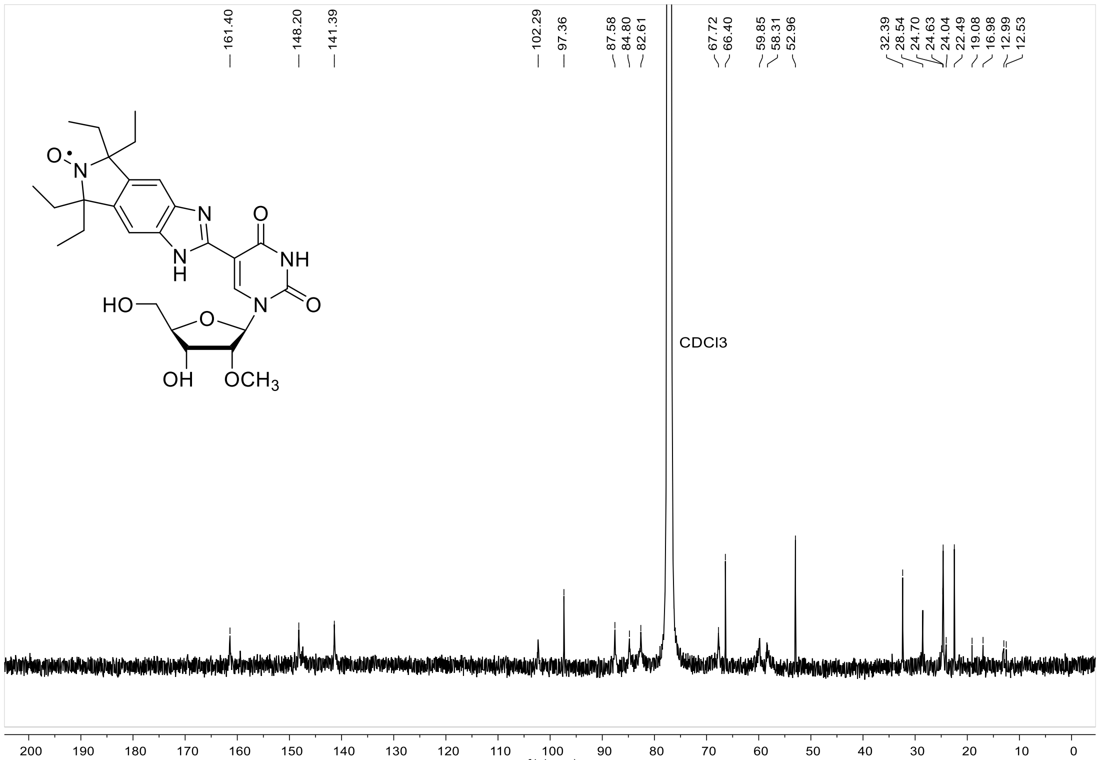

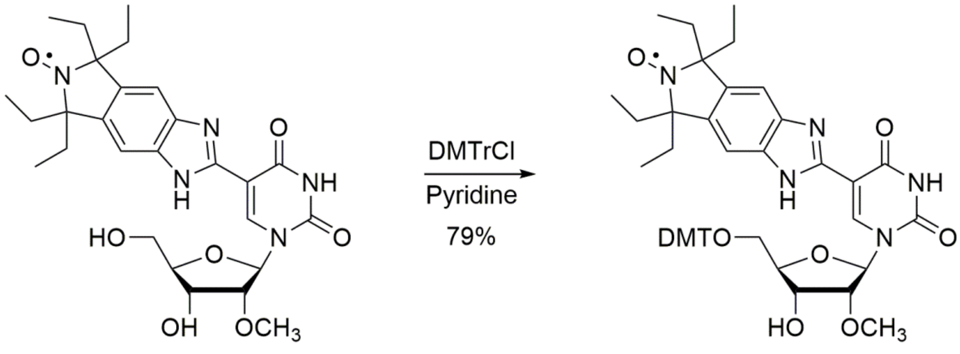

E[Im]Um

7

Nucleoside 7. **E[Im]Um** (80.0 mg, 0.1474 mmol), DMTrCl (99.9 mg, 0.2948 mmol) and DMAP (1.8 mg, 0.01474 mmol) were weighed into a round bottom flask and kept *in vacuo* for 12 h. Pyridine (2.0 mL) was added and the solution was stirred at 23 °C for 3 h. MeOH (100 µL) was added and the solvent was removed *in vacuo*, the residue purified by silica gel column chromatography with a gradient elution (CH_2Cl_2:MeOH; 100:0 to 97:2.5 + 0.5% Et_3N; column prepared in 99.5% CH_2Cl_2 + 0.5% Et_3N) to give tritylated compound **7** as a yellow solid (90.0 mg, 79.0%).

^{1}H NMR ($CDCl_3$): δ 11.34 (br s), 8.94 (br s), 7.46 (br s), 7.39 (br s), 7.19 (br s), 7.07 (br s), 6.77 (br s), 6.07 (br s), 4.49 (br s), 4.15 (br s), 3.80 (br s), 3.69 (br s), 3.58 (br s), 2.88 (br s), 2.86 (br s), 2.08 (br s), 1.29 (br s), 1.20 (br s), 0.91 (br s).

^{13}C NMR ($CDCl_3$) δ 163.19, 158.47, 158.39, 149.74, 144.72, 141.35, 136.00, 135.79, 130.28, 128.38, 128.23, 127.77, 126.73, 126.63, 113.50, 113.14, 113.09, 103.85, 88.81, 86.85, 84.12, 83.46, 82.92, 82.55, 80.71, 77.76, 70.57, 69.23, 64.18, 62.60, 60.50, 59.23, 55.42, 55.33, 45.71, 29.77, 21.17, 14.30, 10.03.

HR-ESI-MS $(M + H)^+$: calcd. for $C_{48}H_{55}N_5O_9$ 845.3994, found 845.3994.

^{1}H NMR spectrum of 7

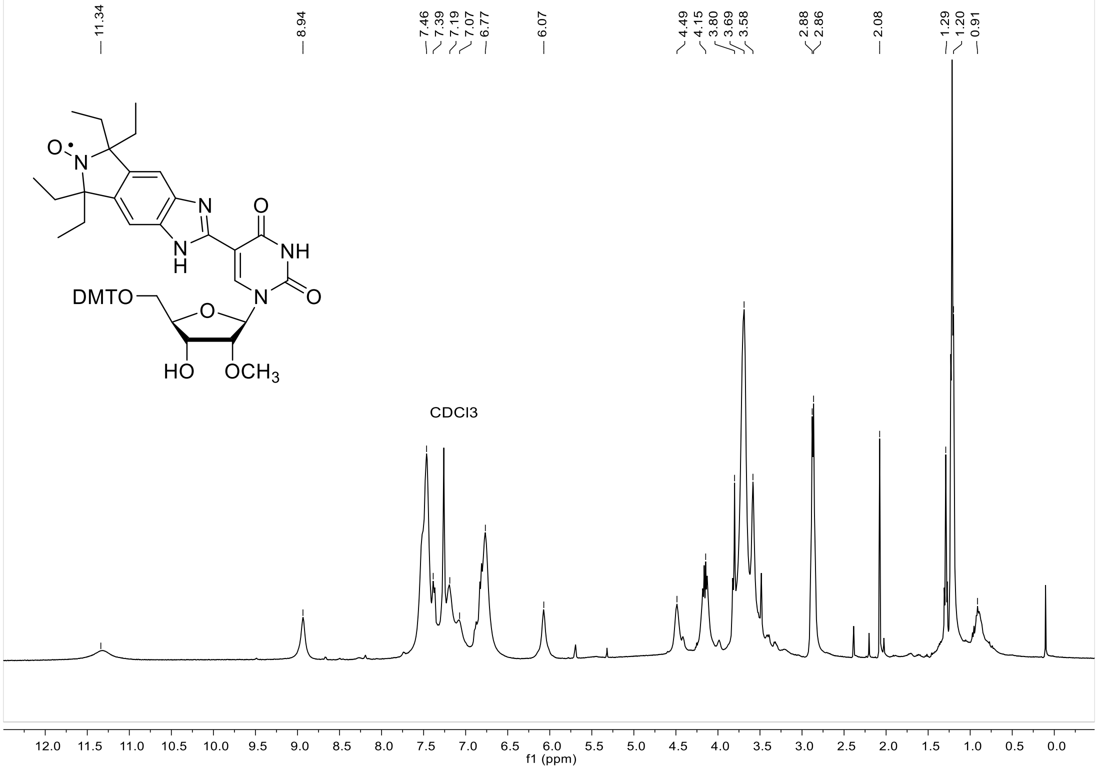

^{13}C NMR spectrum of 7

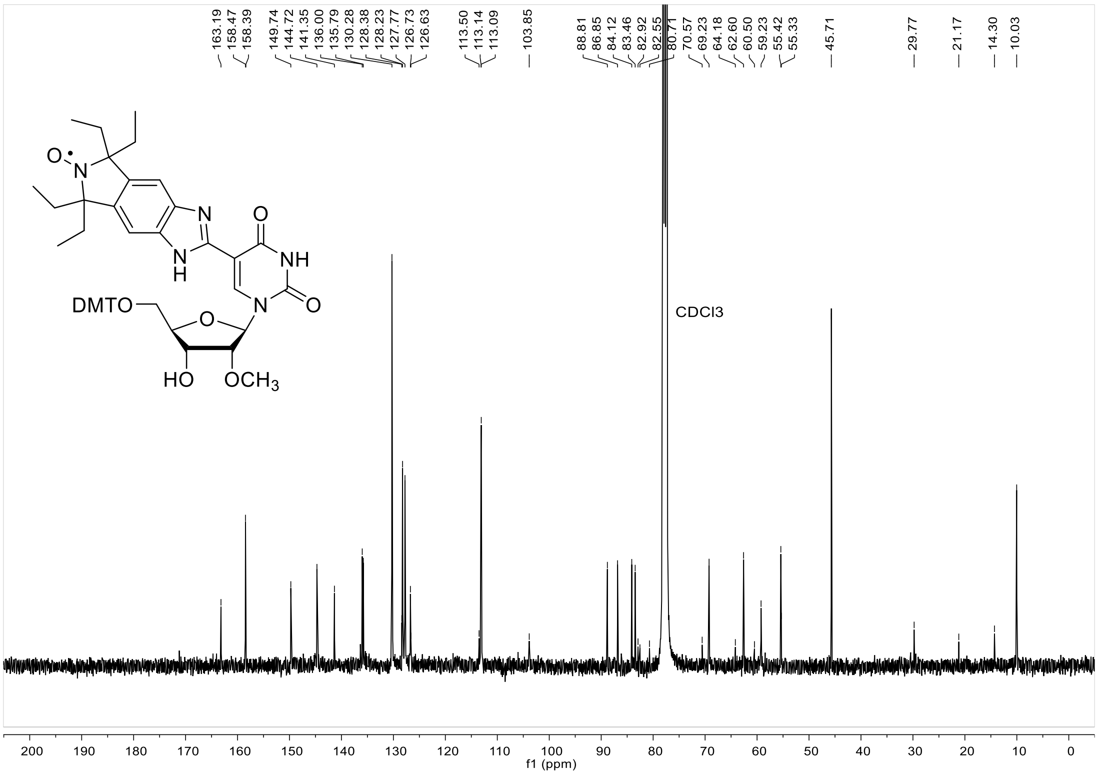

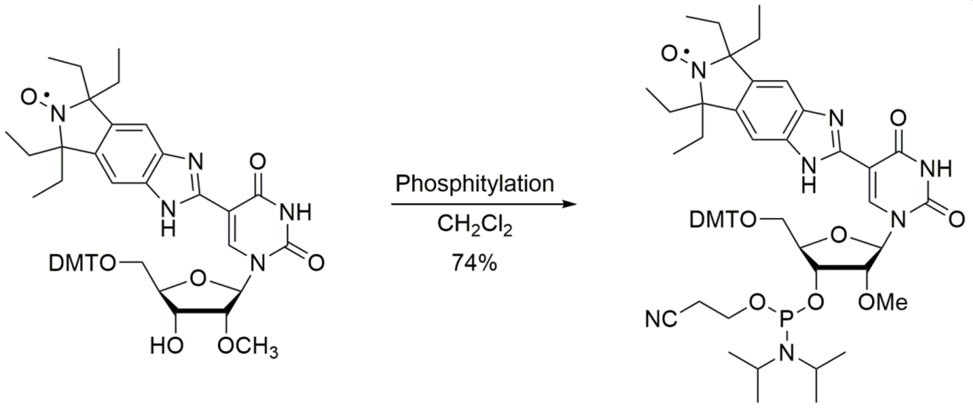

7 8

Phosphoramidite 8. Diisopropyl ammonium tetrazolide (30.0 mg, 0.17 mmol) and nitroxide **7** (100.0 mg, 0.12 mmol) were dissolved in pyridine (2 mL), the solvent evaporated *in vacuo* and the residue kept *in vacuo* for 17 h. CH_2Cl_2 (3.0 mL) and 2-cyanoethyl N,N,N',N'-tetraisopropyl phosphoramidite (107.0 mg, 0.36 mmol) were subsequently added. The reaction mixture was stirred at 23 °C for 16 h, diluted with CH_2Cl_2 (10 mL) and washed successively with saturated aq. $NaHCO_3$ (3 x 10 mL) and saturated aq. NaCl (2 x 10 mL). The combined organic layers were dried over anhydrous Na_2SO_4, filtered and concentrated *in vacuo*. The phosphoramidite was purified by dissolving the residue in CH_2Cl_2 (0.5 mL) and precipitation by addition of petroleum ether (50 mL). The solvent was decanted and the operation repeated three times to furnish phosphoramidite **8** as a yellowish solid (60 mg, 48%), which was used for RNA synthesis without further purification.

^{1}H NMR ($CDCl_3$): δ 11.06 (br s), 9.01 (br s), 8.92 (br s), 7.45 (br s), 6.76 (br s), 6.07 (br s), 5.99 (br s), 4.63 (br s), 4.47 (br s), 4.33 (br s), 4.20 (br s), 3.79 (br s), 3.70 (br s), 3.61 (br s), 3.58 (br s), 3.43 (br s), 2.65 (br s), 2.39 (br s), 1.52 (br s), 1.40 (br s), 1.39 (br s), 1.34 (br s), 1.33 (br s), 1.31 (br s), 1.29 (br s), 1.28 (br s), 1.25 (br s), 1.20 (br s), 1.18 (br s), 1.17 (br s), 1.08 (br s), 1.06 (br s), 0.91 (br s), 0.90 (br s), 0.88 (br s).

^{13}P NMR ($CDCl_3$): δ 150.63, 150.60.

HR-ESI-MS $(M + H)^+$: calcd. for $C_{57}H_{72}N_7O_{10}P$ 1045.5078, found 1045.5125.

^{1}H NMR spectrum of 8

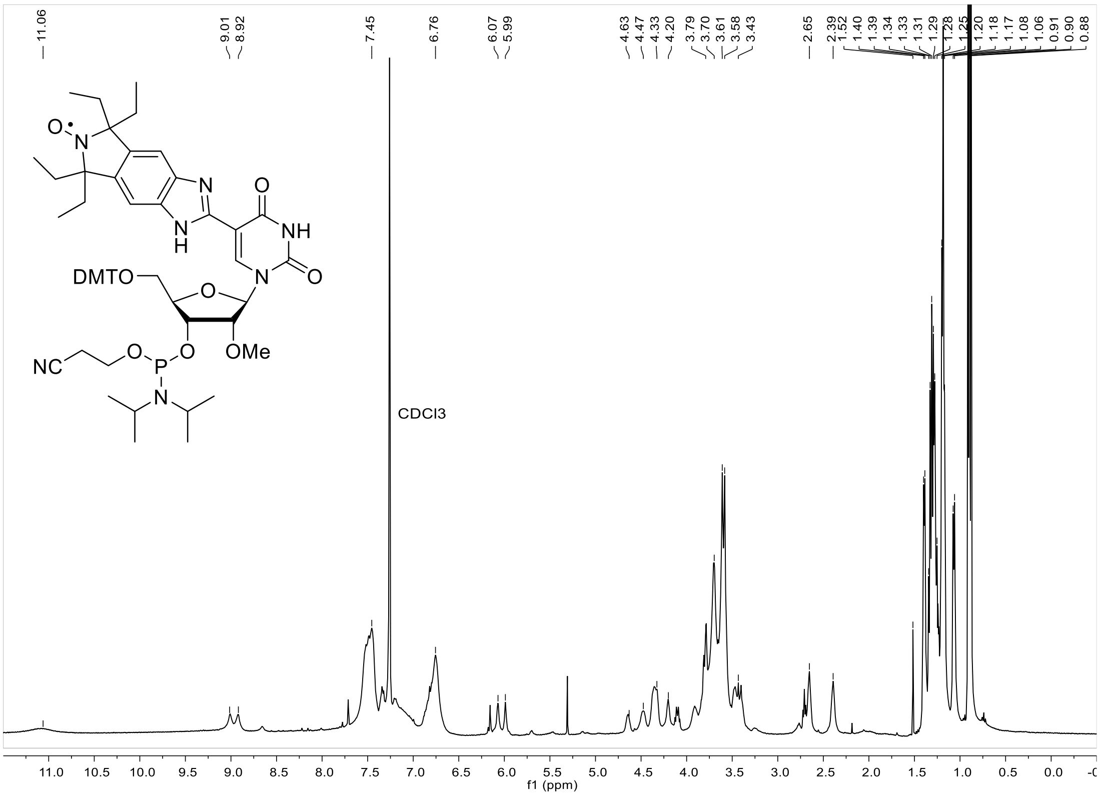

^{31}P NMR spectrum of 8

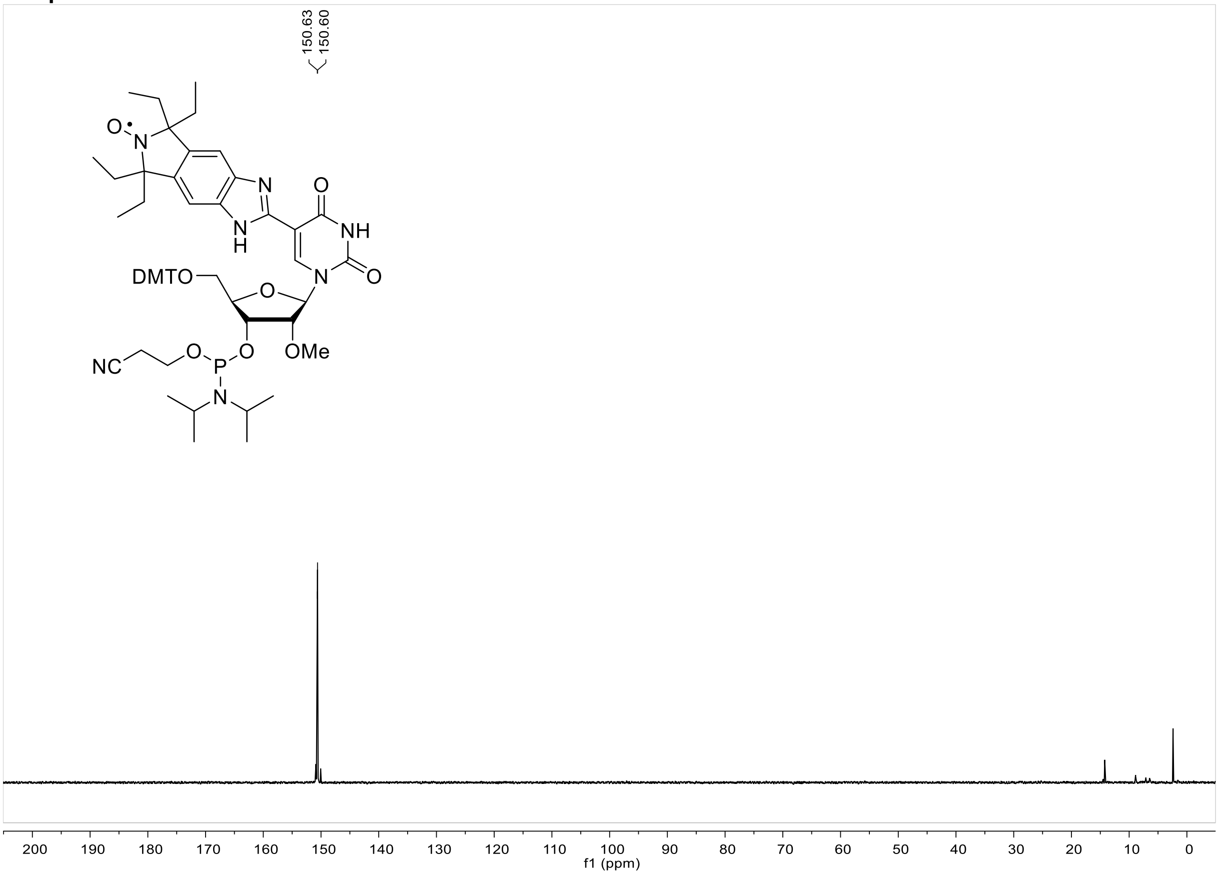

MALDI-TOF MS analyses of oligoribonucleotides

The incorporation of **E**^{Im}**Um** into oligoribonucleotides was confirmed by MALDI-TOF MS analysis. The instrument was calibrated with an external standard prior to measurements. The calculated and observed monoisotopic masses of oligoribonucleotides are listed below in **Table S1**.

Table S1. Monoisotopic masses of oligoribonucleotides.

S. No.	Sequence	Monoisotopic mass (M+H) (calculated)	Monoisotopic mass (M+H) (observed)
1.	5'-G**E**^{Im}**Um**C GAC GGA AGU CGA CAG UA	6765.125	6765.823
2.	5′- UAC **E**^{Im}**Um**GU CGA CUU CCG UCG AC	6576.024	6576.362

MALDI-TOF mass spectrum of oligoribonucleotide 5'-G**E**^{Im}**Um**C GAC GGA AGU CGA CAG UA (calcd. 6765.125, obs. 6765.823):

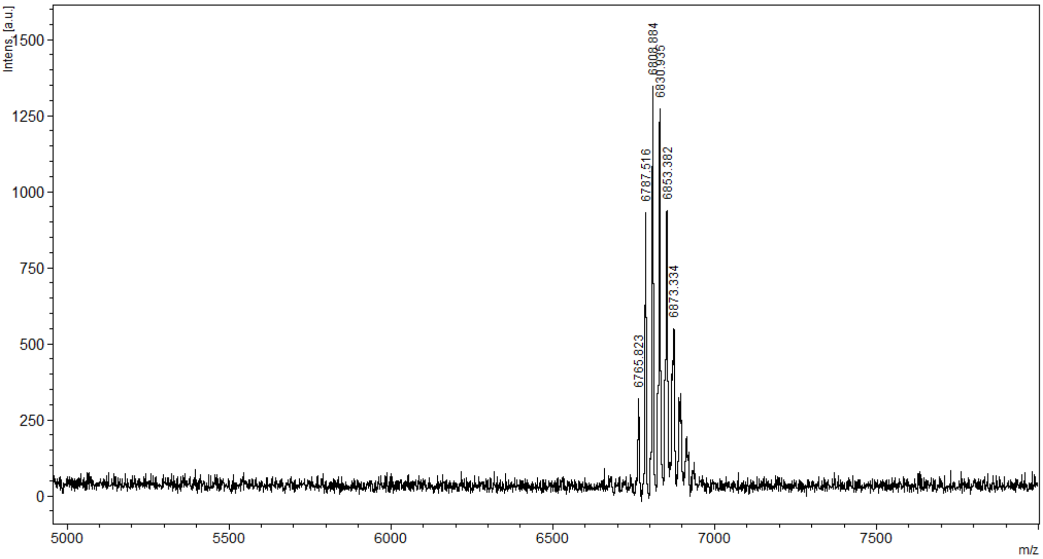

MALDI-TOF mass spectrum of oligoribonucleotide 5'- UAC **E**^{Im}**Um**GU CGA CUU CCG UCG AC (calcd. - 6576.024, obs. 6576.362):

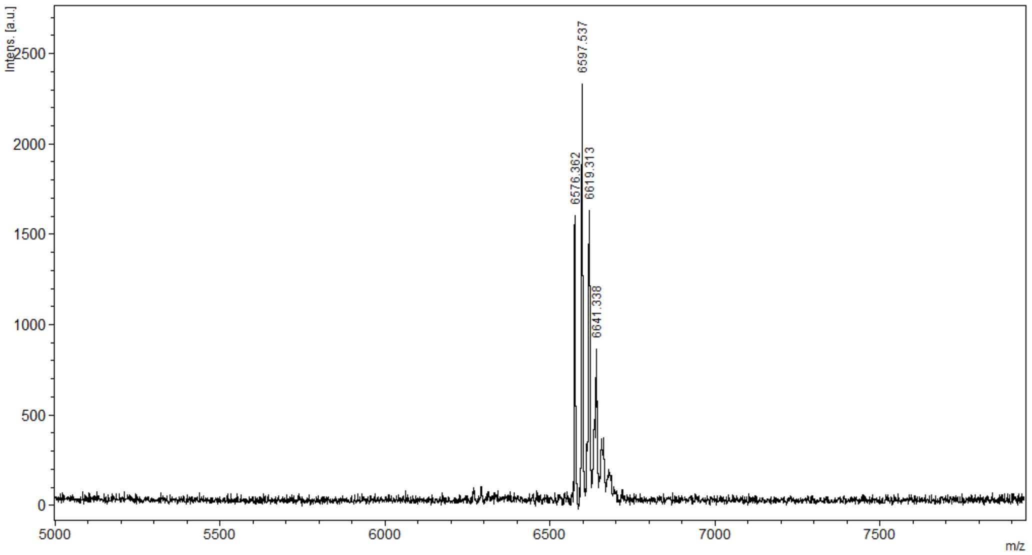

Thermal denaturation experiments

To determine if the **E^{Im}Um** affected the stability of RNA duplexes, the thermal denaturation curves of both unmodified and spin-labeled oligomers were determined and compared. The RNA oligonucleotides (4.0 nmol of each strand) were dissolved in a phosphate buffer (100 µL; 10 mM phosphate, 100 mM NaCl, 0.1 mM Na_2EDTA, pH 7.0), annealed and diluted to 1.0 mL with the phosphate buffer and degassed with argon. The samples were heated up from 20 °C to 90 °C (1.0 °C/min) and absorbance at 260 nm was recorded at 1.0 °C intervals (**Figure S1**). Incorporation of two **E^{Im}Um** modifications into the 20-mer oligoribonucleotide stabilized the duplex by ca. 4.0 °C.

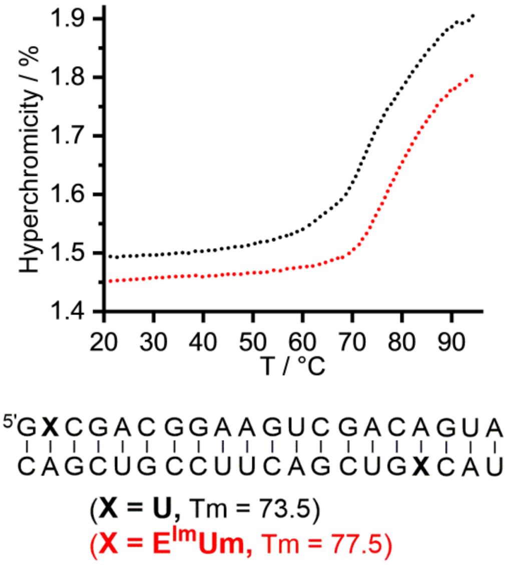

Figure S1. Thermal denaturation curves for **E^{Im}Um**-containing oligoribonucleotides. Black trace: unmodified duplex; red trace: **E^{Im}Um**-modified duplex.

List of the RNA duplexes

In this work the following RNA duplexes were used:

Table S2. Sequences of the RNA duplexes.

RNA	Sequence
**RNA I**	5′-G**E^{Im}Um**C GAC GGA AGU CGA C A G UA-3′<br>3′-C A G CUG CCU UCA GCU G**E^{Im}Um**C AU-5′
**RNA II**	5′-GUC GAC GGA AGU CGA C A G UA-3′<br>3′-CAG CUG CCU UCA GCU G**E^{Im}Um**C AU-5′
**RNA III**	5′-U GCG AUG **E-TU**UA UCU AGA UA A CAU CGC-3′<br>3′-CGC UAC A AU AGA UCU AU**E-TU** GUA GCG U-5′
**RNA IV**	5′-U GCG AUG U**E-TU**A UCU AGA U A A CAU CGC-3′<br>3′-CGC UAC A A U AGA UCU A**E-TU**U GUA GCG U-5′
**RNA V**	5′-GAC CUC GCA UCG UG-3′<br>3′-CUG GAG CGU AGC AC-5′

Preparation of the samples for *in vitro* EPR spectroscopy

In vitro samples for pulsed EPR spectroscopy were prepared at a 100 µM concentration of the doubly spin-labeled RNA duplexes in PNE buffer (10 mM phosphate buffer pH 7.0, 100 mM NaCl, 0.1 mM EDTA) containing 20% v:v glycerol as glass-forming agent.
For the samples in cytoplasmic extract or in concentrated protein solutions (200 mg/mL lysozyme or bovine serum albumin prepared in water/glycerol 8:2 v:v), a lyophilized aliquot of a 100 µM stock solutions of the duplexes in PNE buffer was re-dissolved at a final duplex concentration of 100 µM; in the case of the cytoplasmic extract (*vide infra*), glycerol was added to a final concentration of 20% v:v. All the aforementioned solutions were prepared using DEPC-treated water.
The samples for Q- and X-band EPR spectroscopy were loaded in 1.1 mm ID × 1.6 mm OD or 1.75 mm ID × 2.80 mm OD Suprasil capillaries, respectively, and flash frozen in liquid nitrogen; typical volumes were 5 µL for Q-band samples and 20 µL for X-band samples.
A sample for room-temperature continuous-wave X-band EPR spectroscopy of RNA II was prepared at a 100 µM concentration of the duplex in PNE buffer and loaded in a 20 µL BLAUBRAND® intraMark micropipette.

Preparation of the cytoplasmic extract

The cytoplasmic extract was prepared according to Serber *et al.*[6] Briefly, approximately 500 oocytes were transferred to XB buffer (100 mM KCl, 0.1 mM $CaCl_2$, 1 mM $MgCl_2$, 10 mM K^+-HEPES, 50 mM sucrose) supplemented with 2% wt/vol cysteine and swirled for approximately 30 minutes. The oocytes were subsequently washed first with ORi buffer and then with CSF-XB buffer (XB buffer containing 2 mM $MgCl_2$ and 5 mM EGTA). After having transferred the oocytes into a 2 mL polypropylene microcentrifuge tube, the excess buffer was removed by pipetting; cytochalasin B, leupeptin, pepstatin A and chymostatin were added at a final concentration of 10 µg/mL. The oocytes were subsequently packed by centrifugation at 400 *g* and 4°C for 1 minute. The excess buffer was removed, and the cells were crushed by centrifugation at 16100 *g* and 4°C for 1 hour. The crude interphase extract was eventually removed by piercing the side of the tube with a needle, aliquoted, shock-frozen in liquid nitrogen and stored at -80°C.
Estimation of the protein concentration in the extract was performed using a colorimetric assay (Sigma Aldrich's Advanced Protein Assay Reagent, #57697). The determined values lie around 20 mg/mL; in comparison, the total protein concentration in *Xenopus lævis* oocytes is expected to be about 125 mg/mL assuming a cellular volume of 1 µL.[7]

Internalization of the RNA duplexes inside *Xenopus lævis* oocytes and preparation of the in-cell EPR samples

For the preparation of the samples for the in-cell study, the doubly spin-labeled RNA duplexes were microinjected into mature, defolliculated *Xenopus lævis* oocytes at the stage V/VI. The oocytes were injected with 50.6 nL of a concentrated (0.4 mM – 1.0 mM) stock solution of the RNAs in aqueous buffer. The microinjection always took place starting from the animal hemisphere slightly above the vegetal hemisphere and pointing the needle towards this latter; the exogenous RNA is therefore expected to be delivered to the cytoplasm. The amount of microinjected oocytes was typically 10 for Q-band EPR samples and 50 for pulsed X-band EPR samples. After incubation of the injected oocytes at 16°C in ORi buffer (5 mM HEPES pH = 7.6, 110 mM NaCl, 5 mM KCl, 2 mM $CaCl_2$, 1 mM $MgCl_2$) for a period of one hour, the cells were loaded into an EPR quartz capillary (1.4 mm ID × 1.6 mm OD for Q-band EPR samples, 1.75 mm ID × 2.80 mm OD for pulsed X-band EPR samples). Immediately after preparation, the samples were shock-frozen in liquid nitrogen. The in-cell EPR samples were afterwards stored in liquid nitrogen. The number of oocytes inside the active volume of the used EPR resonators (*vide infra*) is approximately 3 for Q-band samples and 6 for X-band samples.
The sample for room-temperature continuous-wave X-band EPR spectroscopy was prepared in an analogous way by microinjecting 50 oocytes with 50.6 nL of a 1.0 mM solution of RNA II; the cells were loaded into a 1.4 mm ID × 1.6 mm OD EPR quartz capillary and the sample was measured immediately after its preparation.

X-band room-temperature CW-EPR spectroscopy

The *in vitro* X-band CW-EPR spectrum of RNA II (**Figure S2A**, black trace) was recorded at room temperature on a Bruker Elexsys E500 spectrometer equipped with a Bruker ER4122SHQE resonator carrying a quartz Dewar insert. The 20 µL micropipette was inserted for the purpose of the measurement inside a 2 mm ID × 3 mm OD quartz capillary. Relevant acquisition parameters were: microwave power = 2 mW, modulation amplitude = 1.5 G, conversion time = 81.92 ms, time constant = 20.48 ms, sweep time = 83.89 s. In order to verify the stability of the shielded nitroxide inside cells, the in-cell X-band CW-EPR spectrum of RNA II (**Figure S2A**, red trace) was recorded at room temperature on a Bruker Elexsys E500 spectrometer equipped with a Bruker ER4102ST resonator carrying a quartz Dewar insert. The 1.4 mm ID × 1.6 mm OD quartz capillary was inserted for the purpose of the measurement inside a 2 mm ID × 3 mm OD quartz capillary. Relevant acquisition parameters were: microwave power = 2 mW, modulation amplitude = 2.5 G, conversion time = 81.92 ms, time constant = 20.48 ms, sweep time = 83.89 s. Each individual scan was saved and used to evaluate the dependence of the signal intensity with the incubation time (**Figure S2B**).

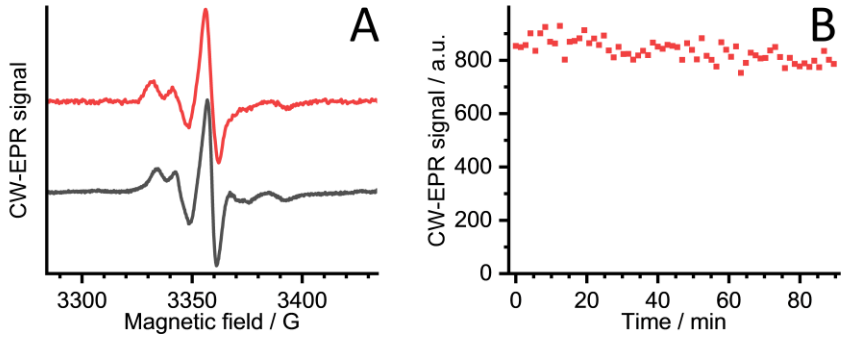

Figure S2. X-band room-temperature CW-EPR spectrum of RNA II *in vitro* (**A**, black trace) and inside cells (**A**, red trace) and dependence of the in-cell signal intensity with the incubation time (**B**).

The measurements confirm the stability of the shielded nitroxide in the intracellular environment. The comparison between the spectra plotted in **Figure S2A** shows furthermore that no significant strand dissociation takes place inside cells.

Instrumentation and general experimental conditions for X- and Q-band pulsed EPR spectroscopy

X-band pulsed EPR experiments were performed at 50 K using a home-built spectrometer equipped with an ER 4118X-MS-3 resonator, a 1 kW microwave amplifier (117X, Applied Systems Engineering Inc.), an Oxford CF935 cryostat and an Oxford ITC503 temperature controller. For all the experiments a shot repetition time of 5.1 ms was used.
Q-band pulsed EPR experiments were performed at 50 K on a Bruker Elexsys E580 spectrometer equipped with an EN 5107D2 resonator, a 150 W microwave amplifier (187Ka, Applied Systems Engineering Inc.), an Oxford CF935 cryostat and an Oxford ITC503 temperature controller. For all the experiments a shot repetition time of 5.1 ms was used.

Echo-detected field-sweep and 2-pulse echo decay experiments at X- and Q-band

In vitro- and in-cell samples of RNA I (**Table S2**) were characterized by X- and Q-band pulsed EPR spectroscopy.
Echo-detected field sweep (EDFS-EPR) experiments (**Figures S3A**, **S3C**) were performed by recording the amplitude of the spin echo generated by a π/2 – τ – π – τ – *echo* sequence while sweeping the magnetic field. The length of the π/2 and π pulses was set to 16 ns and 32 ns, respectively, and the inter-pulse delay τ was set to 200 ns. The spin echo was integrated over its whole width, using a 100 ns-wide range. A two-step phase cycle[8] (x)x was applied.
2-pulse echo decay experiments (**Figures S3B**, **S3D**) were performed at the maximum of the EPR spectrum with the same sequence used for the echo-detected field sweep experiments and varying the length of the inter-pulse delay τ starting from a minimum value of 200 ns.

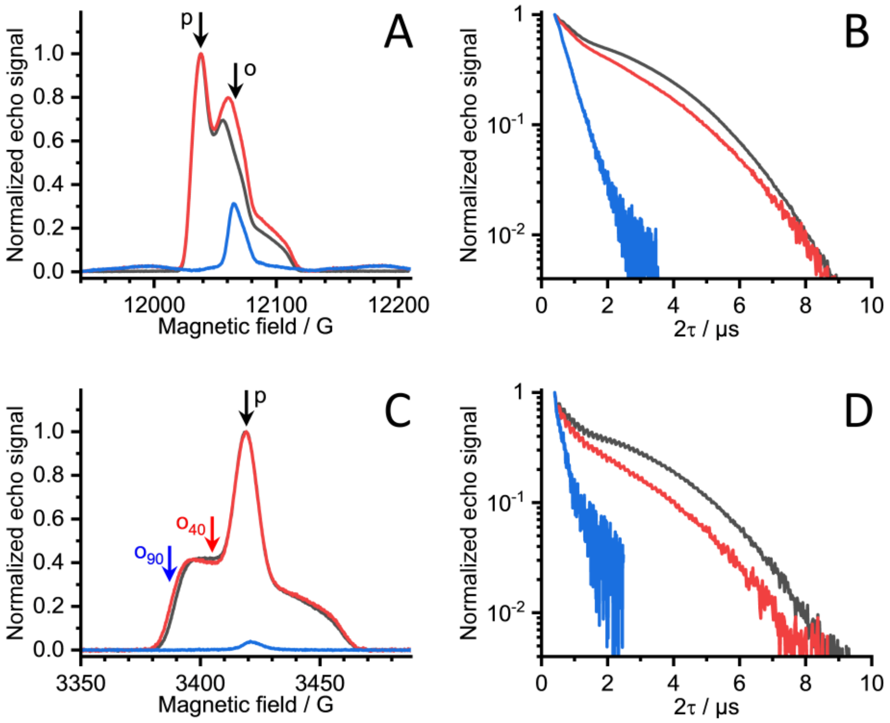

Figure S3. Q-band (upper row) and X-band (lower row) EDFS-EPR spectra (left) and 2-pulse echo decay traces (right) of RNA I *in vitro* (black traces) and in cell (red traces). The blue traces show the endogenous signal of the oocytes; for the Q-band measurement the amplitude of this background signal was scaled to the intensity of the Mn^{2+} component of the spectrum of the injected oocytes, whereas for the X-band measurement this internal scaling could not be performed due to the absence of detectable Mn^{2+} signals. On the EDFS-EPR spectra (A, C) the positions of the pump (p) and observe (o) pulses for PELDOR spectroscopy are indicated; for Q-band spectroscopy $\nu_o = \nu_p$ - 80 MHz, for X-band spectroscopy the two extremes are given (o_{40}: $\nu_o = \nu_p$ + 40 MHz, o_{90}: $\nu_o = \nu_p$ + 90 MHz). The 2-pulse echo decay traces (B, D) were recorded at field values corresponding to the maximum of the respective EDFS-EPR spectra.

We also collected the background signal coming from the oocytes (blue traces in **Figure S3**). The background consists of a broad component, originating from the Mn^{2+} endogenously present in the cells[9] and visible especially at Q-band frequencies owing to the narrowing of the $m_S = -½ \rightarrow +½$ transitions with respect to X-band frequencies, and of a narrow component in the g ~ 2 region, which – according to its nutation behavior – is assigned to a S = ½ spin species. Because the phase memory time of this latter (blue traces in **Figures S3B, S3D**) is much shorter compared to the typical delays used for the PELDOR experiments (*vide infra*), no contribution to the echo of the observed spins is expected. As the overlap between the excitation profile of the pump pulse (FWHM = 67 MHz, corresponding to approximately 24 G, at both X- and Q-band frequencies) and the two components of the background signal is not significant compared to the overlap with the spectral profile of the nitroxide, we also rule out an involvement of the endogenous paramagnetic centers in the observed decrease of the inter-spin distance upon internalization of the RNA duplexes inside the cells.

Q-band PELDOR spectroscopy of RNA I

Q-band PELDOR experiments on RNA I (**Table S2**) were performed using the 4-pulse sequence[10]
$\pi/2 - \tau_1 - \pi - \tau_1{+}t - \pi_p - \tau_2{-}t - \pi - \tau_2 - echo$
whereby eleven traces with τ_1 increments of 2 ns were added, typically starting from a τ_1 value of 300 ns.
A two-step phase cycling[8] (x)yy was applied to the detection sequence. For the pump inversion pulse π_p a 12-ns rectangular pulse generated from a second, incoherent microwave source was used; the position of this pulse was set to match the maximum of the EPR spectrum. The remaining three pulses were also rectangular, all with a length of 22 ns, and had a frequency offset of -80 MHz with respect to the pump pulse (**Figure S3A**).
The PELDOR traces were analyzed using the model-free approach implemented in the MATLAB toolbox DeerAnalysis 2019.[11]

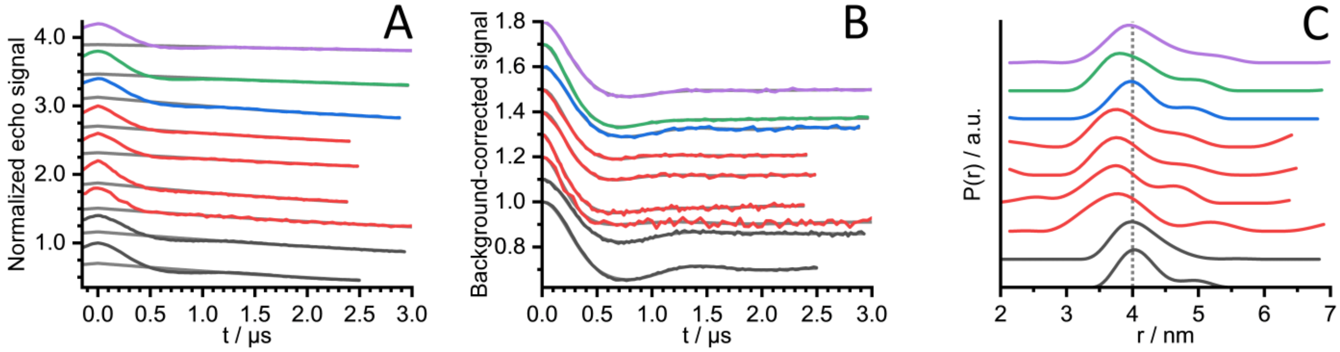

Figure S4. Individual Q-band PELDOR traces of RNA I *in vitro* (black traces), in cell (red traces), in cell extract (blue trace), in 200 mg/mL lysozyme (green trace) and in 200 mg/ml BSA (purple trace). **A**: raw data (colored traces) and intermolecular contributions (grey traces). **B**: intramolecular contributions (colored traces) and fits obtained by Tikhonov regularization (grey traces). **C**: distance probability distributions. Multiple traces, where present, show results obtained from different samples.

For the PELDOR traces shown in **Figure 2A** a validation of the analysis procedure was performed in order to confirm the statistical significance of the reduction of the inter-spin distance upon internalization of RNA I inside cells; the results are shown in **Figure S5**.

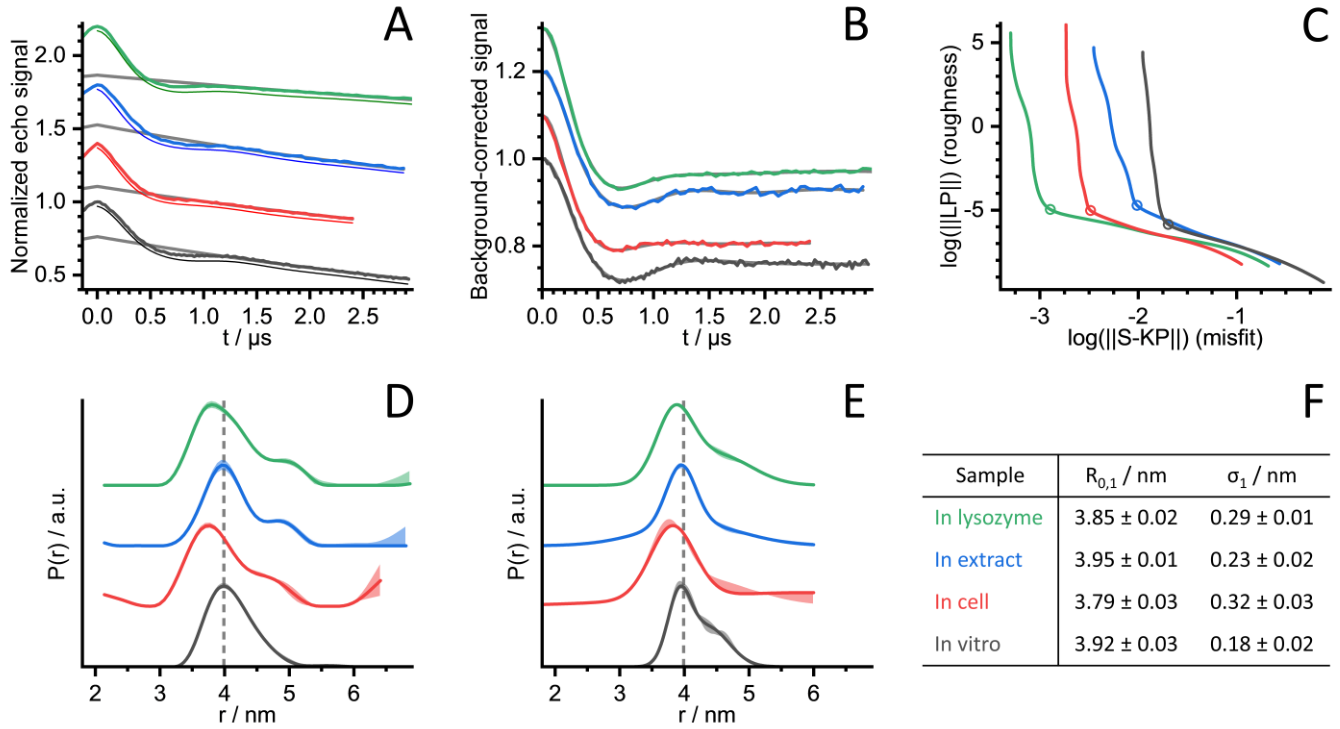

Sample	$R_{0,1}$ / nm	σ_1 / nm
In lysozyme	3.85 ± 0.02	0.29 ± 0.01
In extract	3.95 ± 0.01	0.23 ± 0.02
In cell	3.79 ± 0.03	0.32 ± 0.03
In vitro	3.92 ± 0.03	0.18 ± 0.02

Figure S5. Validation of the analysis procedure for representative PELDOR traces of RNA I. **A**: raw data (thick colored traces), intermolecular contributions (grey traces) and results of the model-based fits (thin colored lines). **B**: intramolecular contributions (colored traces) and fits obtained by model-free analysis (grey traces). **C**: L curves related to the model-free analysis of the traces displayed in panel **B**; the empty circles highlight the chosen values of the regularization parameter α. **D**: Distance probability distributions obtained by Tikhonov regularization and uncertainty ranges evaluated by varying the extent of the region for the fit of the intermolecular contribution. **E**: Distance probability distributions obtained by a 2-Gaussian model-based fit of the traces displayed in panel **A** and corresponding uncertainty ranges. **F**: Parameters of the major component for the model-based fits and corresponding uncertainty ranges.

The validation of the results of the model-free analysis (**Figure S5D**) was performed using the built-in validation tool of DeerAnalysis. The region for the fit of the intermolecular contribution, whose end always coincides with the end of the PELDOR traces, was initially set to the full length of the non-oscillatory part of the raw data and systematically decreased over 50 steps till a minimum length of 750 ns was reached.

The model-based analysis of the data (**Figure S5E**) was performed with a home-written MATLAB-based program that uses a downhill simplex approach to minimize the root-mean-square deviation to the experimental raw data of a calculated time trace $V(t) = V_{intra}(t) \cdot V_{inter}(t)$ corresponding to a two-Gaussian distance probability distribution and an exponentially decaying intramolecular signal; the inclusion of this latter in the simulated data overcomes the issues generated by an incorrect *a priori* processing of the experimental data. For each trace 10000 optimization runs were performed using randomly generated starting points; the 50 runs giving the best fit of the experimental traces were used to estimate the range of variation of the distance probability distribution, displayed as colored areas in **Figure S5E**, and of the fitting parameters.

Additional components in the distance probability distributions in the region around 4.5 – 5.0 nm could be observed for all the tested conditions with both the model-free and model-based analyses of the PELDOR traces (**Figures S4C, S5E**). The blunt ends in the RNA could have caused some end-to-end stacking of the duplexes,[12] and this could be related to the aforementioned long distances. However, a more definitive assignation is not possible because distances above 4.6 nm cannot be determined accurately with a maximum extension of the dipolar evolution window of 3 µs,[13]

X-band PELDOR spectroscopy of RNA I

X-band PELDOR experiments on RNA I (**Table S2**) were performed using the 4-pulse sequence[10]

$\pi/2 - \tau_1 - \pi - \tau_1{+}t - \pi_p - \tau_2{-}t - \pi - \tau_2 - echo$

whereby τ_1 was incremented in 8-ns steps from 300 ns to 364 ns to average out proton nuclear modulation. All the pulses were generated from the same microwave source; an eight-step phase cycling[8] $(x)y[x_p]\bar{y}$ was applied.[14]

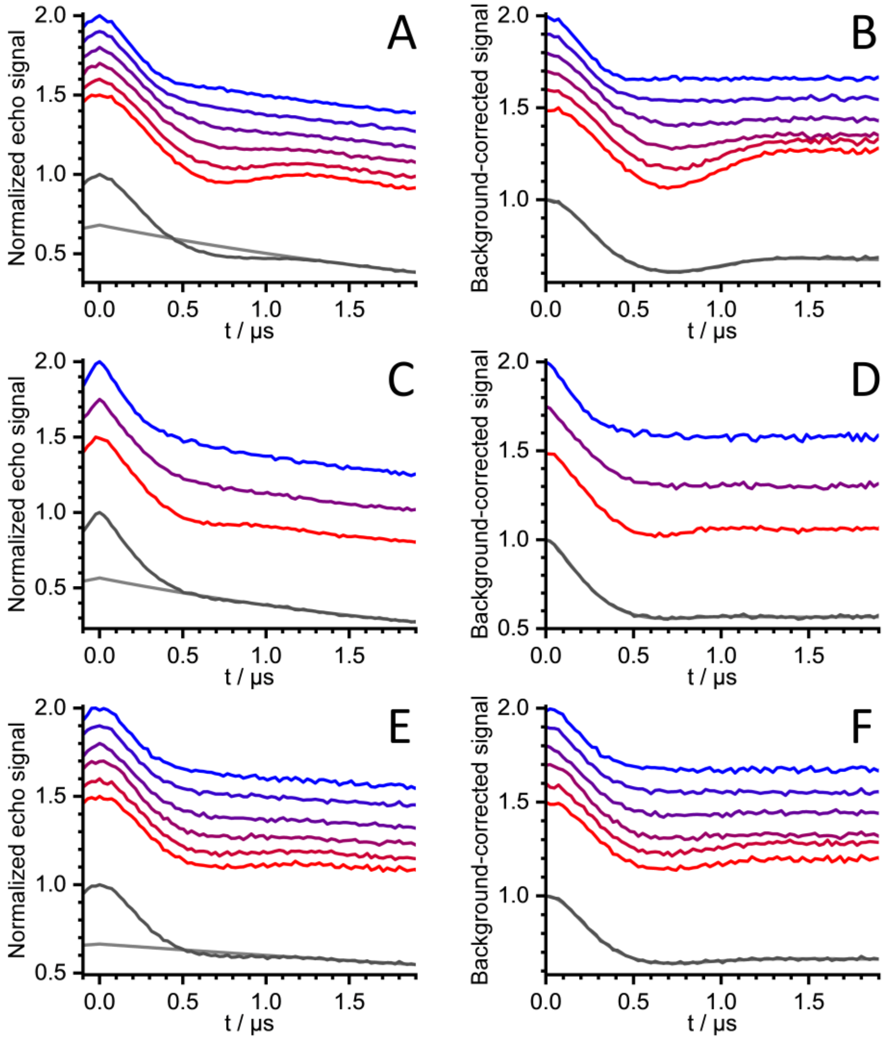

Figure S6. X-band PELDOR data of RNA I *in vitro* (**A**, **B**), in cell (**C**, **D**) and in 200 mg/mL lysozyme (**E**, **F**). Left: raw data, right: intramolecular contributions. Frequency offsets (see also **Figure S3C**): 40 MHz (red traces) – 90 MHz (blue traces) in steps of 10 MHz (*in vitro*, in lysozyme) or 25 MHz (in cell). Black traces: sum of the traces measured at different frequency offsets; grey traces: intermolecular contributions.

For the pump inversion pulse π_p a Gaussian pulse[15] with a FWHM of 8.78 ns and a length of 28 ns was used; the position of this pulse was set to match the maximum of the EPR spectrum. The remaining three pulses were also Gaussian, all with a FWHM of 23.4 ns and a length of 72 ns, and had a frequency offset with respect to the pump pulse between 40 MHz (**Figure S6**, red traces) and 90 MHz (**Figure S6**, blue traces); for the samples *in vitro* and in lysozyme 6 traces were collected with a 10 MHz step, whereas for the in-cell sample 3 traces were collected with a 25 MHz step.

The observed dependence of the intramolecular part of the PELDOR signal on the frequency offset between the pump and observe pulses for the *in vitro* sample (**Figure S6B**) reflects the presence of angular correlations between the spin labels, confirming their restricted mobility with respect to the RNA backbone.[16] This effect is substantially washed out both in lysozyme (**Figure S6D**) and in cells (**Figure S6F**), indicating a more heterogeneous conformational ensemble of the RNA duplex in these environments.

In order to extract the distance probability distributions, the traces measured at different frequency offsets were summed[17] (**Figure S6**, black traces) and the resulting traces were analyzed by Tikhonov regularization using the MATLAB toolbox DeerAnalysis 2019 (**Figure S7**).[11]

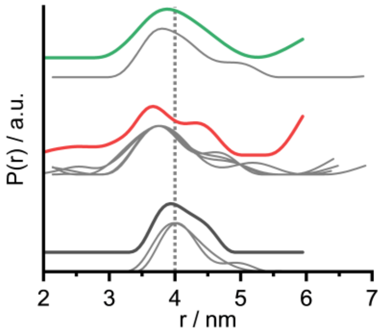

Figure S7. Comparison of the distance probability distributions obtained from the averaged X-band PELDOR data (colored thick traces: black (*in vitro*), red (in cell), green (in 200 mg/mL lysozyme); the original traces are reported in **Figure S6**, black traces) and the Q-band PELDOR data (grey thin traces; see **Figures S4C** and **2B**. Multiple traces, where present, show results obtained from different samples.).

The very good comparison between the distance probability distributions obtained from the averaged X-band PELDOR data (**Figure S7**, thick colored lines) and the Q-band PELDOR data (**Figure S7**, thin grey lines) confirms the absence of angular correlation effects in these latter.

Q-band PELDOR spectroscopy of the E-TU-labeled RNA duplexes

Q-band PELDOR experiments were performed on the 24mer duplexes RNA III and RNA IV (**Figure S8B**) in vitro, in cell and in 200 mg/mL lysozyme to rule out the possibility that the spin label and/or the sequence of the duplex plays an explicit role on the detected change of the inter-spin distance upon internalization inside cells. The used RNA sequences carry furthermore 5'-U overhangs to prevent the end-to-end stacking of the duplexes.[12]

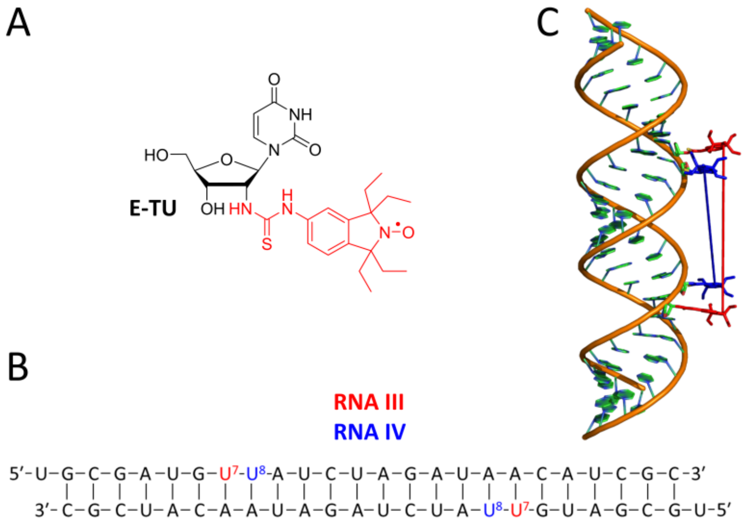

Figure S8. A. Structure of the E-TU spin label. **B**. Sequence of the 24mer RNA duplex; the spin-labeled nucleotides are in red for RNA III and in blue for RNA IV. **C**. Model of the RNA duplex containing the E-TU labels.

The duplex are labeled with the tetraethyl-shielded, thiourea-based spin label E-TU[2] (**Figure S8A**). The PELDOR experiments (**Figure S9**) were performed using the same settings described for RNA I.

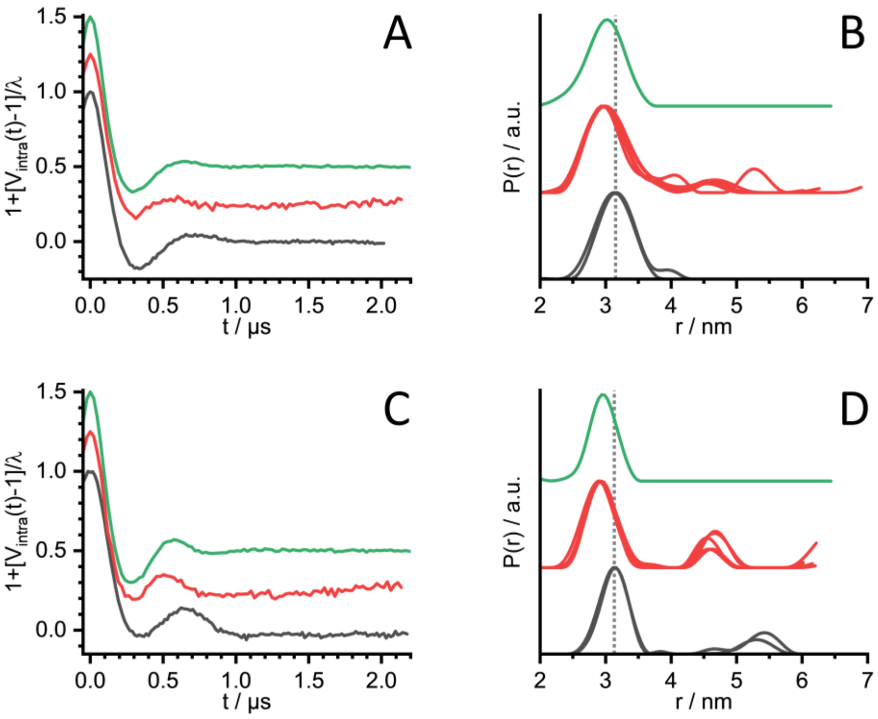

Figure S9. Q-band 4-pulse PELDOR background-subtracted data normalized by the modulation depth (**A**, **C**) and the corresponding distance probability distributions obtained by model-free analysis (**B**, **D**) for the duplex RNAs III (upper row) and RNA IV (lower row) in a buffered solution (10 mM phosphate pH 7.0, 100 mM NaCl, 0.1 mM EDTA; black traces), in *Xenopus lævis* oocytes (red traces) and in a 200 mg/mL lysozyme solution (green trace). Multiple traces, where present, show results obtained from different samples. The original traces are reported in **Figure S10**.

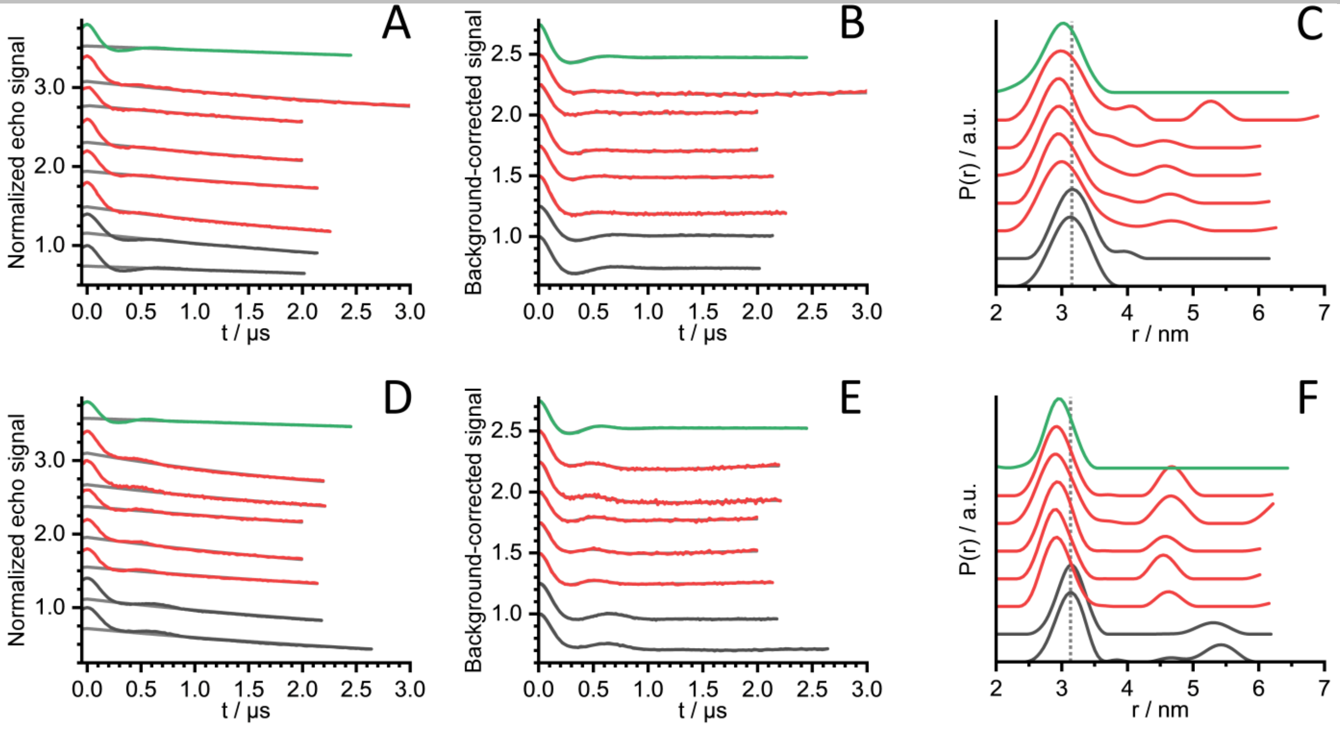

Figure S10. Individual Q-band PELDOR traces of RNA III (upper row) and RNA IV (lower row) *in vitro* (black traces), in cell (red traces) and in 200 mg/mL lysozyme (green trace). **A, D**: raw data (colored traces) and intermolecular contributions (grey traces). **B, E**: intramolecular contributions (colored traces) and fits obtained by Tikhonov regularization (grey traces). **C, F**: distance probability distributions. Multiple traces, where present, show results obtained from different samples.

Native polyacrylamide gel electrophoresis (PAGE)

Native polyacrylamide gel electrophoresis (PAGE) of dsRNA samples having different NaCl/lysozyme ratios was performed to test the role of electrostatic interactions in the binding of lysozyme to RNA. The 14mer RNA V (**Table S2**) was used for this experiment.
A 0.75 mm-thick gel was prepared by polymerizing a 18% 19:1 acrylamide/bis-acrylamide solution (BioRad, Inc.) containing 1× TBE buffer (100 mM TRIS, 100 mM boric acid, 2 mM EDTA) with ammonium persulfate and tetramethylethylenediamine (TEMED); for the preparation of the gel, DEPC-treated water was used.
The samples, each containing 2 µg of dsRNA, were loaded using a glycerol-containing buffer. A Bio-Rad Mini-PROTEAN Tetra Cell equipment was used to perform the electrophoresis.
After running under mild conditions (120 V for 2 hours), the gel was stained with a 3× GelRed solution in water (Biotium, Inc.) containing 100 mM NaCl.

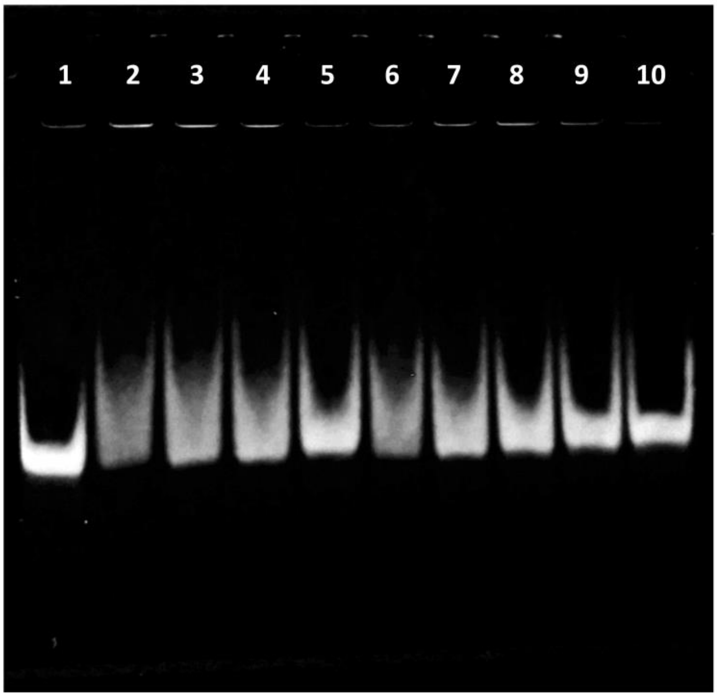

Figure S11. Native PAGE (18% acrylamide) of RNA V for different NaCl/lysozyme ratios. **Lane 1**: 2 µg dsRNA. **Lanes 2 – 5**: 2 µg dsRNA, 150 µg lysozyme, 0 – 58 – 117 – 292 µg NaCl. **Lanes 6 – 9**: 2 µg dsRNA, 75 µg lysozyme, 0 – 58 – 117 – 292 µg NaCl. **Lane 10**: 2 µg dsRNA, 292 µg NaCl.

Molecular dynamics simulations

As starting structure for the molecular dynamics simulations, an unlabeled double-stranded RNA A-helix with the sequence shown in **Figure 1B** of the main paper (RNA I; see also **Table S2**) was generated using the make-na server (*structure.usc.edu/make-na/server.html*). An NMR solution structure (PDB: 1E8L[18]) was used as starting structure for hen egg-white lysozyme. The protein structure was subsequently protonated at pH = 7.0 using the propKa implementation of the Maestro Schroedinger software package (*https://www.schrodinger.com/maestro*). For simulations without lysozyme [-LYZ], the RNA duplex was inserted into a simulation box, solvated with the TIP3P[19] or TIP4P-D water model[20] and Na^+ and Cl^- ions[21] at 150 mM concentration. For simulations with lysozyme [+LYZ], 20 lysozyme proteins were inserted randomly into the box in non-overlapping configurations and solvated. Molecular dynamics simulations were performed using the Gromacs simulation package [v. 2016.5][22] with the leap-frog algorithm and an integration step of 2 fs. For the RNA duplex, the ff99bsc0χOL3[23] and the DESRES force field[24] were used. Lysozyme was described by the Amber99SB*-ILDN-Q protein force field.[25] **Table S3** provides a detailed list of all simulation setups performed.

Table S3. Properties of the molecular dynamics simulation systems. LYZ: lysozyme; R1-R6: simulation runs 1-6; n(dsRNA): number of dsRNA in the simulation box; n(LYZ): number of lysozymes in the simulation box; c(LYZ): (approximate) lysozyme concentration; L: edge length of cubic simulation box; t_{total}: total simulation time. Force field references are given in the section "Molecular dynamics simulations".

System name	n(dsRNA)	n(LYZ)	c(LYZ) [mg/mL]	Atoms	L [nm]	t_{total} [ns]
OL3/TIP3P [-LYZ]	1	0	0	91,139	9.7	1,104
OL3/TIP4P-D [-LYZ]				121,768		1,112
DESRES/TIP4P-D [-LYZ]				121,768		1,663
OL3/TIP4P-D [+LYZ, R1]	1	20	~200	305,874	13.3	2,787
OL3/TIP4P-D [+LYZ, R2]				306,342		591
OL3/TIP4P-D [+LYZ, R3]				306,386		594
OL3/TIP4P-D [+LYZ, R4]				306,470		597
OL3/TIP4P-D [+LYZ, R5]				306,270		591
OL3/TIP4P-D [+LYZ, R6]				305,882		595

After energy minimization up to local convergence, all simulations were equilibrated in an NVT ensemble with the velocity rescaling thermostat (τ = 1 ps)[26] for 100 ps before equilibration in an NPT ensemble with the Parrinello-Rahman barostat (τ = 5 ps)[27] for 5 ns. Production runs were performed with the same parameters as the NPT equilibration. Non-bonded electrostatic interactions were computed with the Particle Mesh Ewald method.[28] Lennard-Jones and real-space electrostatic interactions were calculated up to a cutoff of 1.0 nm. A shift function was used for the Lennard-Jones interactions. The lengths of atomic bonds to hydrogens were constrained with LINCS.[29]

We tested that the different combinations of RNA, protein, and water force fields capture the dsRNA structure. As shown earlier, the combination of TIP4P-D for water and Amber99SB*-ILDN-Q for proteins results in an accurate description of crowded solutions of lysozyme and other proteins.[30] The description of an isolated RNA helix with OL3 and TIP4P-D is on par with well-established force fields combinations (OL3 RNA+TIP3P and DESRES+TIP4P-D), as judged by the root-mean-square distance (RMSD) of nucleobase heavy atoms to an idealized A-helix (**Figure S12**).

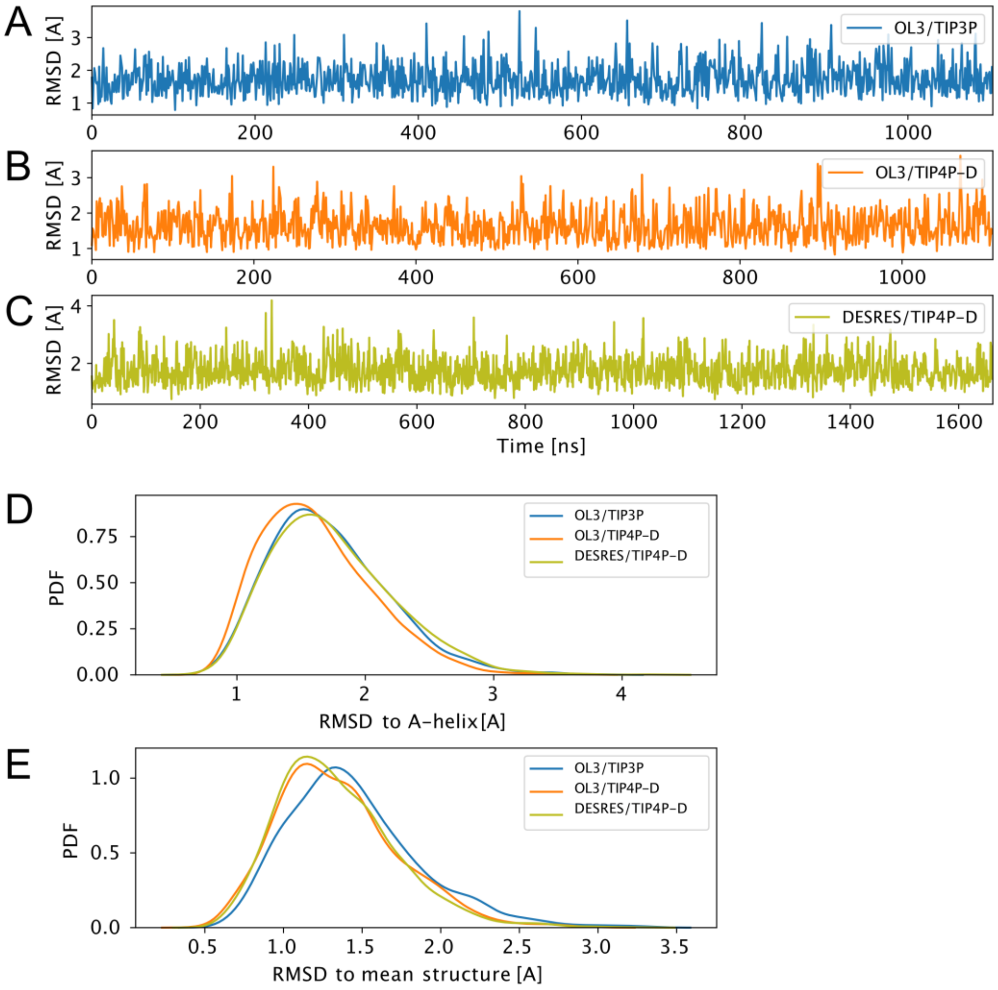

Figure S12. Molecular dynamics simulations of the dsRNA duplex free in solution. (**A-C**) Nucleobase RMSD to ideal A-helix RNA (time traces) for OL3 with TIP3P (**A**) and TIP4P-D (**B**), and for DESRES with TIP4P-D (**C**). (**D,E**) Distributions of the nucleobase RMSD to an ideal A-helix (**D**) and to the mean structure (**E**).

The dsRNA structure is stable over time, as demonstrated by the time traces in **Figure S12** and the structural similarity to the mean structure of the RNA free in solution. The A-type structure of dsRNA is also stable in concentrated lysozyme solution (**Figure S13**). We conclude that the different combinations of RNA, protein, and water force fields preserve the RNA A-helix structure.

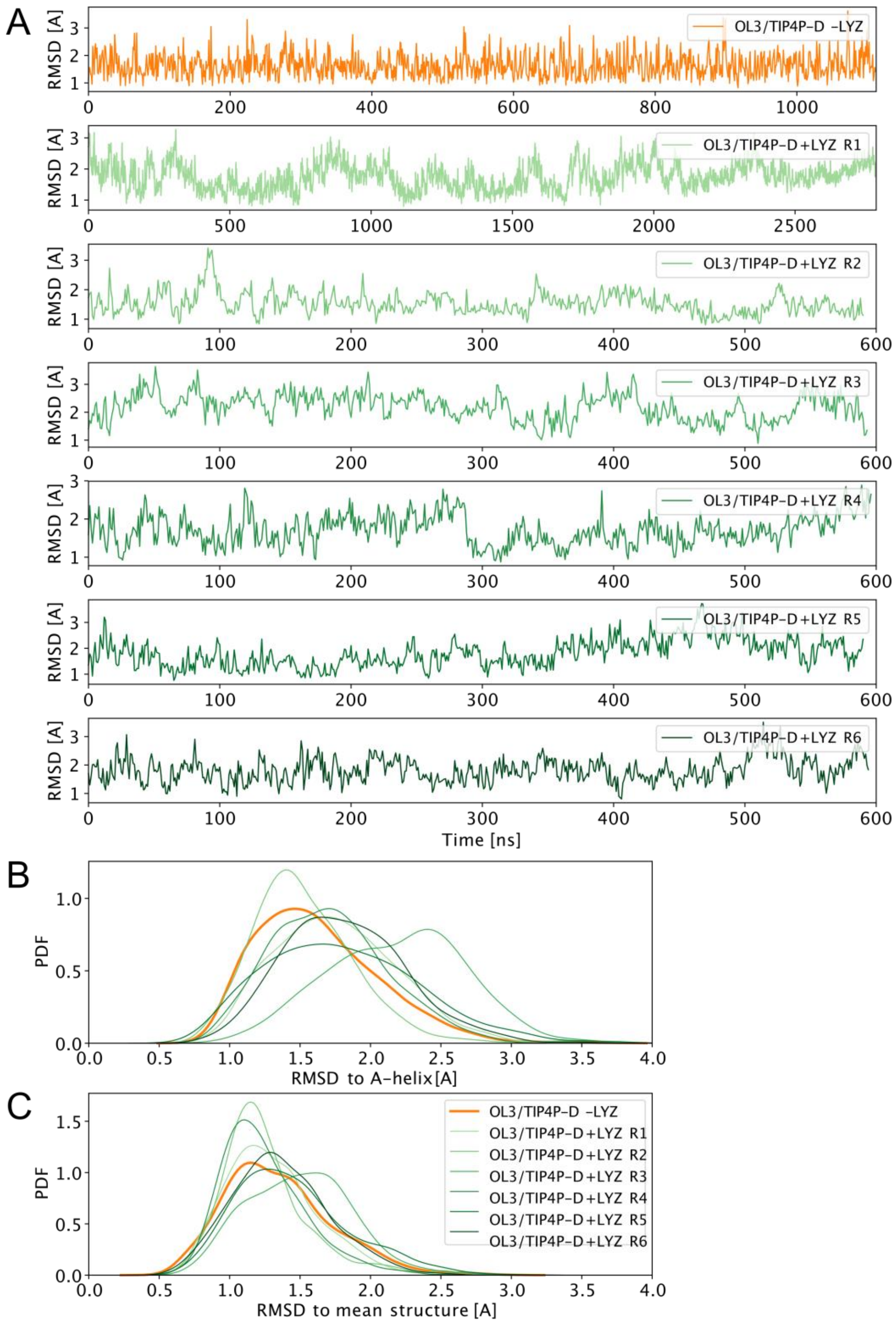

Figure S13. Molecular dynamics simulations of the dsRNA duplex in concentrated solution of lysozyme. (**A**) Time traces of nucleobase RMSD to ideal A-helix RNA in six independent runs. (**B,C**) Probability distribution of nucleobase RMSD to an ideal A-helix (**B**) and to the mean structure across all six simulations (**C**).

SUPPORTING INFORMATION

A systematic comparison between the RNA structures from the molecular dynamics simulations in dilute solution and in lysozyme is performed by means of the analysis of the rigid-body structural parameters[31,32] (**Figure S14**). The analysis confirms that the RNA helix structure is preserved in the presence of lysozyme.

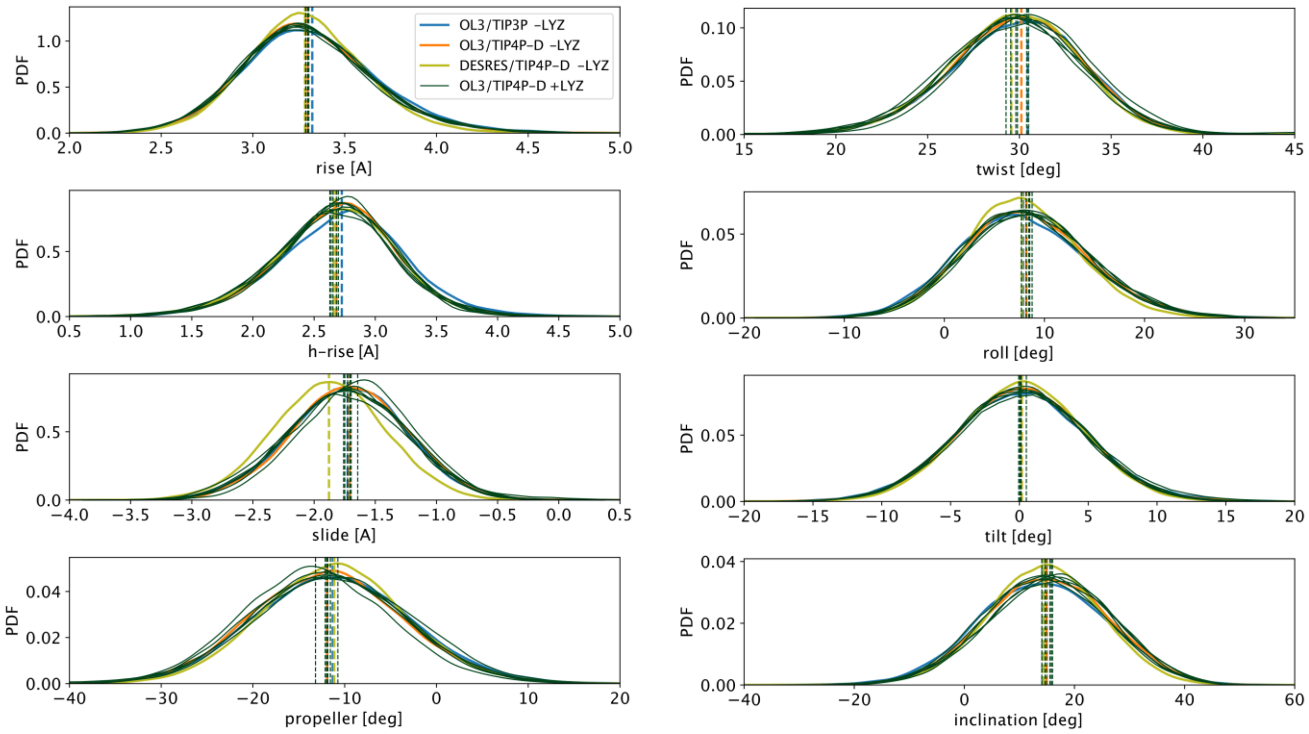

Figure S14. Distribution of rigid-body structural parameters of the RNA from the molecular dynamics simulations with lysozyme [+LYZ] and without lysozyme [-LYZ].

The MD simulations in lysozyme show diverse lysozyme-RNA binding modes, reflecting the nonspecific character of this interaction (**Figure S15**). Differences between the runs (R1-R6) arise from limited sampling of the RNA-protein interactions in the simulation.

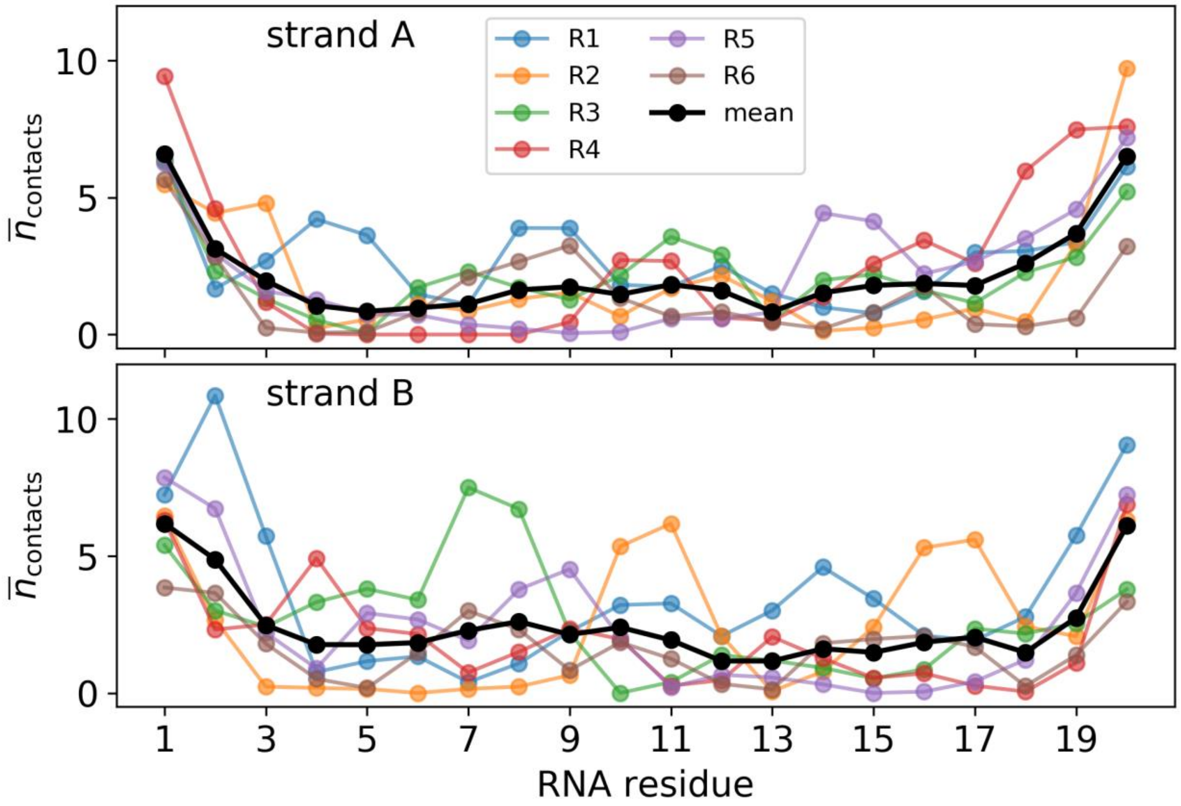

Figure S15. Interactions of lysozyme with strands A and B in the six molecular dynamics simulations (R1-R6) of dsRNA with lysozyme. $\bar{n}_{contacts}$ is defined as the mean number of contacts per simulation snapshot between RNA residues and lysozyme residues. Contacts are determined according to a distance cutoff <1 nm between the RNA residue center-of-mass and protein alpha-carbons. Also shown is the mean across the six runs.

References

[1] C. Höbartner, G. Sicoli, F. Wachowius, D. B. Gophane, S. T. Sigurdsson, *J. Org. Chem.* **2012**, *77*, 7749-7754.
[2] S. Saha, A. P. Jagtap, S. T. Sigurdsson, *Chem. Commun.* **2015**, *51*, 13142-13145.
[3] A. P. Jagtap, I. Krstic, N. C. Kunjir, R. Hänsel, T. F. Prisner, S. T. Sigurdsson, *Free Radic. Res.* **2015**, *49*, 78-85.
[4] D. B. Gophane, S. T. Sigurdsson, *Chem. Commun.* **2013**, *49*, 999-1001.
[5] D. B. Gophane, B. Endeward, T. F. Prisner, S. T. Sigurdsson, *Org. Biomol. Chem.* **2018**, *16*, 816-824.
[6] Z. Serber, P. Selenko, R. Hänsel, S. Reckel, F. Löhr, J. E. Ferrell, G. Wagner, V. Dötsch, *Nat. Protoc.* **2007**, *1*, 2701-2709.
[7] R. L. Hallberg, D. C. Smith, *Dev. Biol.* **1975**, *42*, 40-52.
[8] S. Stoll, B. Kasumaj, *Appl. Magn. Reson.* **2008**, *35*, 15-32.
[9] M. Qi, A. Groß, G. Jeschke, A. Godt, M. Drescher, *J. Am. Chem. Soc.* **2014**, *136*, 15366-15378.
[10] M. Pannier, S. Veit, A. Godt, G. Jeschke, H. W. Spiess, *J. Magn. Reson.* **2000**, *142*, 331-340.
[11] G. Jeschke, V. Chechik, P. Ionita, A. Godt, H. Zimmermann, J. Banham, C. R. Timmel, D. Hilger, H. Jung, *Appl. Magn. Reson.* **2006**, *30*, 473-498.
[12] N. Erlenbach, C. Grünewald, B. Krstic, A. Heckel, T. F. Prisner, *RNA* **2019**, *25*, 239-246.
[13] G. Jeschke, *Annu. Rev. Phys. Chem.* **2012**, *63*, 419-446.
[14] C. E. Tait, S. Stoll, *Phys. Chem. Chem. Phys.* **2016**, *18*, 18470-18485.
[15] M. Teucher, E. Bordignon, *J. Magn. Reson.* **2018**, *296*, 103-111.
[16] O. Schiemann, P. Cekan, D. Margraf, T. F. Prisner, S. T. Sigurdsson, *Angew. Chemie - Int. Ed.* **2009**, *48*, 3292-3295.
[17] A. Marko, D. Margraf, H. Yu, Y. Mu, G. Stock, T. Prisner, *J. Chem. Phys.* **2009**, *130*, 064102.
[18] H. Schwalbe, S. B. Grimshaw, A. Spencer, M. Buck, J. Boyd, C. M. Dobson, C. Redfield, L. J. Smith, *Protein Sci.* **2001**, *10*, 677-688.
[19] W. L. Jorgensen, J. Chandrasekhar, J. D. Madura, R. W. Impey, M. L. Klein, *J. Chem. Phys.* **1983**, *79*, 926-935.
[20] S. Piana, A. G. Donchev, P. Robustelli, D. E. Shaw, *J. Phys. Chem. B* **2015**, *119*, 5113-5123.
[21] I. S. Joung, T. E. Cheatham, *J. Phys. Chem. B* **2008**, *112*, 9020-9041.
[22] M. J. Abraham, T. Murtola, R. Schulz, S. Páll, J. C. Smith, B. Hess, E. Lindah, *SoftwareX* **2015**, *1-2*, 19-25.
[23] M. Zgarbová, M. Otyepka, J. Šponer, A. Mládek, P. Banáš, T. E. Cheatham, P. Jurečka, *J. Chem. Theory Comput.* **2011**, *7*, 2886-2902.
[24] D. Tan, S. Piana, R. M. Dirks, D. E. Shaw, *Proc. Natl. Acad. Sci.* **2018**, *115*, E1346-E1355.
[25] R. B. Best, D. De Sancho, J. Mittal, *Biophys. J.* **2012**, *102*, 1462-1467.
[26] G. Bussi, D. Donadio, M. Parrinello, *J. Chem. Phys.* **2007**, *126*, 014101.
[27] M. Parrinello, A. Rahman, *J. Appl. Phys.* **1981**, *52*, 7182-7190.
[28] T. Darden, D. York, L. Pedersen, *J. Chem. Phys.* **1993**, *98*, 10089-10092.
[29] B. Hess, H. Bekker, H. J. C. Berendsen, J. G. E. M. Fraaije, *J. Comput. Chem.* **1997**, *18*, 1463-1472.
[30] S. von Bülow, M. Siggel, M. Linke, G. Hummer, *Proc. Natl. Acad. Sci. U. S. A.* **2019**, *116*, 9843-9852.
[31] X. J. Lu, W. K. Olson, *Nucleic Acids Res.* **2003**, *31*, 5108-5121.
[32] R. Kumar, H. Grubmüller, *Bioinformatics* **2015**, *31*, 2583-2585.

Author Contributions

A.C., S.v.B., L.S.S., G.H., S.Th.S. and T.F.P. designed the research; A.C. prepared the samples and performed the PELDOR experiments; S.v.B. and L.S.S. performed the molecular dynamics simulations; D.B.G. synthesized the RNAs labeled with E^{lm}Um; S.S. synthesized the RNAs labeled with E-TU; A.C., S.v.B., L.S.S., G.H., S.Th.S. and T.F.P. analyzed the data and wrote the manuscript; G.H., S.Th.S. and T.F.P. provided funding, supervised and administered the project.
